# Recombination Enhances HIV-1 Envelope Diversity by Facilitating the Survival of Latent Genomic Fragments in the Plasma Virus Population

**DOI:** 10.1371/journal.pcbi.1004625

**Published:** 2015-12-22

**Authors:** Taina T. Immonen, Jessica M. Conway, Ethan O. Romero-Severson, Alan S. Perelson, Thomas Leitner

**Affiliations:** 1 Theoretical Biology and Biophysics, Los Alamos National Laboratory, Los Alamos, New Mexico, United States of America; 2 Department of Mathematics, The Pennsylvania State University, University Park, Pennsylvania, United States of America; University of Zurich, SWITZERLAND

## Abstract

HIV-1 is subject to immune pressure exerted by the host, giving variants that escape the immune response an advantage. Virus released from activated latent cells competes against variants that have continually evolved and adapted to host immune pressure. Nevertheless, there is increasing evidence that virus displaying a signal of latency survives in patient plasma despite having reduced fitness due to long-term immune memory. We investigated the survival of virus with latent envelope genomic fragments by simulating within-host HIV-1 sequence evolution and the cycling of viral lineages in and out of the latent reservoir. Our model incorporates a detailed mutation process including nucleotide substitution, recombination, latent reservoir dynamics, diversifying selection pressure driven by the immune response, and purifying selection pressure asserted by deleterious mutations. We evaluated the ability of our model to capture sequence evolution *in vivo* by comparing our simulated sequences to HIV-1 envelope sequence data from 16 HIV-infected untreated patients. Empirical sequence divergence and diversity measures were qualitatively and quantitatively similar to those of our simulated HIV-1 populations, suggesting that our model invokes realistic trends of HIV-1 genetic evolution. Moreover, reconstructed phylogenies of simulated and patient HIV-1 populations showed similar topological structures. Our simulation results suggest that recombination is a key mechanism facilitating the persistence of virus with latent envelope genomic fragments in the productively infected cell population. Recombination increased the survival probability of latent virus forms approximately 13-fold. Prevalence of virus with latent fragments in productively infected cells was observed in only 2% of simulations when we ignored recombination, while the proportion increased to 27% of simulations when we allowed recombination. We also found that the selection pressures exerted by different fitness landscapes influenced the shape of phylogenies, diversity trends, and survival of virus with latent genomic fragments. Our model predicts that the persistence of latent genomic fragments from multiple different ancestral origins increases sequence diversity in plasma for reasonable fitness landscapes.

## Author Summary

Increasing evidence suggests that HIV-1 released from activated latent cells survives in productively infected cells in patient plasma despite competition against better adapted virus variants that have evolved in response to the host immune pressure. Long-term survival requires that latent virus forms adapt to the host immune response so that they are not outcompeted. We simulated the dynamics of HIV-1 envelope sequence evolution in response to host immune pressure to investigate how virus from activated latent cells can survive despite having reduced fitness compared to the more evolved virus variants in patient plasma. The evolutionary trends of our simulated virus populations followed closely those observed in HIV-1 sequence data from 16 patients. Our simulation results suggest that recombination facilitates the survival of genomic fragments originating from virus activated from latent cells. Our model further predicts that sequence diversity increases with the number of latent genomic fragments from different origins that persist in plasma.

## Introduction

Patients infected with HIV-1 require lifelong highly active antiretroviral therapy (HAART) to suppress infection. Treatment cessation typically leads to HIV viral rebound to pre-therapy levels; the resurgence is thought to be associated with activation of long-lived, latently HIV-infected cells. A cure for HIV therefore requires either clearance of all cells harboring latent virus, or prevention of virus release from the reservoirs after discontinuation of treatment. Increasing evidence suggests that latency plays an integral role throughout the life cycle of the virus. We recently observed that the majority of HIV-1 plasma sequences in two untreated chronically infected patients had accumulated significantly less mutations than expected, suggesting a period of latency during which no replication occurred in the history of these lineages [1]. Moreover, viral variants with reduced evolution consistent with periods of latency are frequently involved in transmission events [2–4]. While recent advances have shed light on the mechanisms leading to the establishment and maintenance of latent reservoirs [5, 6], the prevalence of viral sequences displaying a signal of latency in the replicating population remains enigmatic, especially in the absence of antiviral treatment.

HIV-1 is subject to selection pressure exerted by the immune system; strains that can avoid the immune response have an advantage within a host. The neutralizing antibody response in patient sera is much stronger against virus circulating in infection earlier than contemporaneous variants, with immunological memory persisting for years [7–9]. Virus from activated latent cells is therefore less fit due to long-term immunological memory than variants that have continually evolved in response to the host immune pressure. Yet, we detect a signal of latency in the replicating population, suggesting that virus from activated latent cells persists despite competing against better-adapted contemporaneous variants.

In order to survive, activated latent forms must either have a replicative advantage outweighing immune selection, or quickly acquire escape mutations to evade the immune response. While mutations associated with immune escape decrease the replicative capacity of the virus between 0–24% [10], compensatory mutations can completely restore fitness in some variants [11]. It is thus unknown whether virus from activated latent cells is able to compete against better adapted contemporaneous virus due to higher replicative fitness. To persist in plasma after activation, the virus must adapt to immune pressure before being outcompeted. The virus may adapt by sequentially accumulating escape mutations at recognized epitopes, or by recombining with contemporaneous virus and simultaneously gaining multiple epitopes not recognized by the immune system.

Recombination provides a mechanism to preserve genomic fragments originating from activated latent cells within contemporaneous virus backbones adapted to the immune response. Further recombination events may propagate latent genomic fragments through the replicating plasma population, enhancing the adaptability potential of the virus. In this paper, we investigate the persistence of latent envelope genomic fragments in the replicating plasma population by developing a detailed model of HIV-1 sequence evolution and the cycling of lineages in and out of the latent reservoir.

There has been an increasing focus on modeling the dynamics of the latent reservoir, particularly in the context of delineating the effect of latency on viral blips during treatment, and the re-emergence of infection after treatment failure/interruption [12–16]. A common feature of these models is the cycling of infected cells through a latent compartment, in which they may divide or die before becoming activated and re-joining the productively infected cell population. Our agent-based model integrates the dynamics of the latent reservoir proposed in [12–14], with a sequence evolution framework first introduced in [17, 18], which follows how individual viruses mutate and recombine.

Vijay et al. developed an HIV sequence evolution model of infected patients that incorporates mutation, infection of cells by multiple virions, recombination, fitness selection, and epistatic interactions between multiple loci [17]. Their model, which describes quantitatively the evolution of HIV-1 diversity and divergence in patients, has been applied to a wide variety of questions, including the effect of recombination on sequence diversification [17], the effective population size of HIV-1 [18], the genetic structure of HIV-1 quasispecies and its error threshold [19], and the fraction of progeny viruses that must incorporate a drug treatment target for suppression of productive infection [20].

We extend the sequence evolution model by incorporating a latent compartment in which no evolution takes place, and by explicitly simulating how the virus population interacts with and adapts to the immune response. To evaluate whether our model captures sequence evolution *in vivo*, we compare our simulated sequences to sequences from 16 untreated or unsuccessfully treated patients followed longitudinally from seroconversion [9, 21, 22] in terms of sequence divergence (i.e. average distance of a population of sequences from a founder sequence), diversity (i.e. average pairwise distance between all sequences in a population), and phylogenetic tree shape measures.

## Models

### Model overview

**Fig 1:**
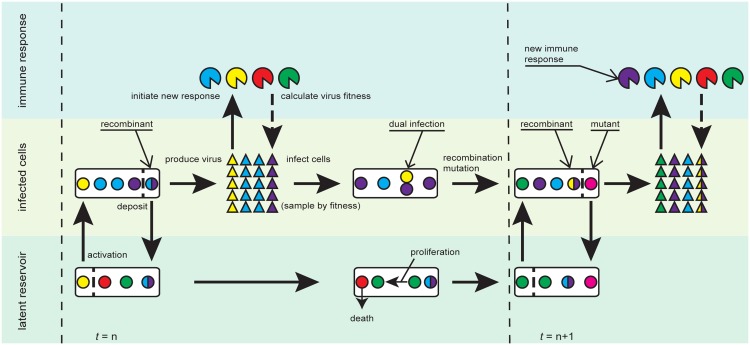
Model schematic. We model interactions between and within three compartments: the latent reservoir, productively infected cells, and the immune response during generation *n*. The circles represent infected cells (both productively infected and latent), the triangles progeny virus, and the pies (circular sectors) the immune response. The colors of the circles and triangles represent different viral epitopes, and the colors in the pies indicate which viral epitopes are recognized by the immune response. Each productively infected cell produces the same number of infectious virus particles. The population of virus is sampled based on fitness to form the next set of productively infected cells, where the fitness of each virus depends on whether it is recognized by the immune response. If a new viral antigen reaches high enough numbers in the plasma, it triggers an immune response. Upon infection a small fraction of cells becomes latent. To mimic this, we assign a small probability to an infected cell moving to the latent reservoir. Also, cells in the latent reservoir have some probability of being activated and joining the replicating population. The viral sequences in productively infected cells are mutated, mimicking events that occur during reverse transcription, and the two parental strains in dually infected cells have some probability of recombining. Cells in the latent reservoir have some probability of dying, and homeostatically proliferate such that the size of the reservoir is maintained.

We developed an agent-based model to simulate within-host HIV-1 sequence evolution after primary HIV-1 infection (PHI), tracking how long each virus lineage has spent in the latent reservoir throughout its history (Fig 1). The model incorporates a detailed mutation process including recombination, latent reservoir dynamics, diversifying selection pressure driven by the immune response, and purifying selection pressure asserted by deleterious mutations. During each generation, a population of virus infects a population of uninfected cells, with infecting viruses chosen according to their relative fitness. Genetic variability is introduced through recombination and substitution during reverse transcription of viral RNAs to proviral DNA in infected cells. Infected cells have a nonzero probability of moving into the latent reservoir, while latent cells have a nonzero probability of dying, proliferating, or becoming activated. Infected cells and activated latent cells produce progeny virus, forming the population of virus infecting the next generation of cells.

The relative fitness of each virus depends on whether it has acquired mutations at invariant sites, which are considered deleterious, and how well it is recognized by the immune system. All virus sequences have multiple epitopes at pre-defined sites, which are presented to the immune system. If an epitope variant reaches a sufficient frequency in the plasma, it elicits an immune response, which remains in memory for the duration of the simulation. The breadth of the immune response is updated each generation by adding any newly recognized epitope variants to immunological memory.

While we simulate infections established by a single founder strain, the model can also accommodate multiple founder strains, which are observed in 20% of heterosexual cases [23]. The simulations were implemented using a computer program written in R [24], and the phylogenies were created using the R package ape (Analyses of Phylogenetics and Evolution) [25]. The algorithm is outlined in the supplement (S1 Text).

### Dynamics of productively infected cells

We initialized the simulation by generating a set of *N*_*V*_ identical virions, where each virion consists of a string of *L* nucleotides (A, U, G, C). While HIV contains two RNA molecules, we considered only a single RNA molecule for each virus to simplify computations. Because recombination between two identical parental strains does not lead to new genetic variants, restricting our simulations to one RNA molecule per virus should have a negligible effect on our simulation results. We chose as the starting sequence the consensus of the first time point sequences, sampled close to seroconversion, of Patient A from the Amsterdam cohort [9]. The sequence is from *env*, and consists of approximately 700 nucleotides.

At each generation, *N*_*C*_ viruses are chosen to infect *N*_*C*_ uninfected cells such that each cell is infected with a single virus. Multiple infection of cells is described below. The probability of selecting a given virus is proportional to its relative fitness, which is a function of the strength of the immune response against it, and the number of deleterious mutations it has acquired. Fitness selection is described in detail below. Upon infection each cell has a small probability *η* of becoming latent and moving to the latent reservoir. The virus in the infected cells that remain productive undergo reverse transcription, where the viral RNA is copied into proviral DNA. This process includes both recombination and substitution.

To simplify computations, we only considered dual infections that lead to recombination. Based on estimates of Josefsson et al. [26], we assumed that 10% of cells are infected with a second virion, ignoring multipe infection with three or more virions. On average half of the progeny virus produced by a cell infected with two virions acquires one RNA molecule from each parent. To simplify computations, we allowed at most one cross-over event per sequence. This simplification is justified because while there are on average 2.8 cross-overs per each HIV-1 genome of 10,000 nucleotides every life cycle [27], our simulated *env* sequences were over 10 times shorter.

For a template switching rate of 3 × 10^−4^ per site per generation [27, 28], the expected number of cells accommodating a recombination event between two different parental strains is *N*_*R*_ = 0.1 × 0.5 × 3 × 10^−4^ × *N*_*C*_ *L*. We incorporated dual infection into the model by sampling *N*_*R*_ additional viruses from the virus population with probability proportional to their relative fitness and adding them to randomly chosen infected cells. The dually infected cells then undergo recombination.

We assumed that cross-over events are distributed uniformly across the genome, selecting the starting strand and the cross-over position ∈ {2,*L* − 1} randomly. The sequence of the starting strand is copied until the cross-over position, the strand is then switched, and the second strand is copied to the end of the sequence. The recombinant sequence replaces the two parent strands, so that all infected cells contain a single viral sequence. The sequence is then mutated according to a general-time-reversible substitution model [29]. We used the nucleotide-specific substitution rates estimated from the sequences of Patient A via maximum likelihood (PAUP*, [30]), which were representative of the rates found for all 16 patients. The proviral DNA of each infected cell is transcribed to viral RNAs, which are released as new virions. Each infected cell produces *P* identical virions, which form the current population of virus from which viruses that infect the next generation of cells are selected.

### Dynamics of the latent reservoir

As the latent reservoir is established early in infection [31, 32], we initialized the reservoir by infecting each of the *N*_*L*_ cells with the same founder virus used to infect the replicating population. During effective antiretroviral treatment, the reservoir’s half-life has been estimated to range from 4 to 44 months [33–36]. Assuming that the size of the latent reservoir, *N*_*L*_, remains constant during untreated chronic infection, we set the probability of an infected cell becoming latent equal to the decay rate of the latent reservoir. Each generation, we randomly select which (if any) infected cells become latent.

For simplicity, we assumed homogeneous activation and death rates, randomly selecting which cells in the latent compartment die or become activated each generation. Activated latent cells join the population of productively infected cells and produce progeny virus. We assumed that the latent reservoir is maintained through homeostatic proliferation [12, 13, 37], which we simulated by randomly duplicating cells to replace those lost to death and activation to keep the size of the latent pool constant. Specifically, if the size of latent reservoir *N*_*L*_ after the addition of any newly latent cells and the removal of any activated or dead cells is less than the target size $N_{L}^{*}$, we randomly duplicate $N_{L}^{*}-N_{L}$ of the remaining cells in the latent compartment. No proliferation takes place if the size of the latent reservoir exceeds $N_{L}^{*}$.

The model tracks how many generations each cell spends in the latent reservoir before being activated, with progeny viruses retaining the information for the duration of the lineage. Because viruses that have been latent for different amounts of time can recombine, we track latency separately at each site in the genome. If a virus that has never been latent recombines with a virus that has been latent for *n* generations, we consider the recombinant progeny a latent virus form, with the portion of its genome acquired from the non-latent parent recorded as having been latent for zero generations, and the portion acquired from the latent parent as having been latent *n* generations. At the end of the simulation, we know the position and age of every latent genomic fragment for each virus sequence. We are interested in the persistence of sequences that contain at least one latent genomic fragment consisting of at least one site originally from a latent ancestor.

### Immune selection and fitness

The immune response against antigens with recognized epitopes consists of two major arms; neutralization of a pathogen via antibodies, and killing of infected cells by cell-mediated immune responses. Because we simulated the viral envelope, we focused primarily on the antibody response, assuming that 1) variants with a recognized epitope are less fit than those able to evade the immune response, and 2) once an epitope is recognized, it is retained in memory for all time. The immune response imposes diversifying selection pressure on the virus population, resulting in sequential escape at each epitope. A virus variant not recognized by the immune system has a fitness advantage, and starts to take over the virus population, eventually eliciting an immune response, and thus selection for new escape variants.

We chose the location of *N*_*E*_ epitopes randomly in the envelope fragment that we are simulating. Epitopes are approximately 30 nucleotides in length, and may be non-contiguous and overlapping [38]. As synonymous mutations at the nucleotide level have no effect at the amino acid level, we ignored third-codon positions, and assumed that the epitopes are 20 nucleotides long and non-overlapping.

Serum from HIV-1 infected individuals does not neutralize contemporaneous virus, but rather virus that dominated the population 3 to 6 months earlier [7]. To model this behavior, we imposed an immune response against a new escape variant not when it first appeared but after it had risen in frequency in the plasma, and increased the strength of the subsequent response gradually over time.

We began the immune response against the virus after 30 generations. Every generation thereafter, we determined which epitope variants had reached a sufficient frequency, *f*, in the plasma, and initiated an immune response against them. The epitopes were stored in immunological memory for the duration of the simulation, and all virus variants containing them had a fitness disadvantage. For simplicity, we assumed that to be recognized the nucleotide sequence of a virus epitope must match perfectly with the sequence of a stored epitope. Note that virus may be recognized at multiple epitopes simultaneously.

Because antibody responses mature over time, we assumed that the fitness cost to the virus imposed by the immune response to epitope *i* of variant *j* increases linearly until it reaches some maximum value $c_{i}^{*}$. Thus, the fitness cost of epitope *i* of variant *j* is given by

$$\begin{array}{c} c_{ij}\left(t\right)=min\left\{c_{i}^{*},c_{i}^{*}\left(t-t_{ij}^{0}\right)/d\right\}\delta_{ij},t\geq t_{ij}^{0}, \end{array}$$

where $t_{ij}^{0}$ is the time when the immune response against epitope *i* of variant *j* is introduced, *d* is the time it takes to reach full potency, and *δ*_*ij*_ is the Dirac delta function, where *δ*_*ij*_ = 1 when epitope *i* of variant *j* is recognized, and *δ*_*ij*_ = 0 when it is not recognized.

The strength of the immune response against a particular epitope depends in part on how accessible it is to antibodies/killer T-cells. While escape mutations appear shortly after seroconversion in HIV-1/SIV infection at some epitopes [10, 39], it may take years to see evidence of selection at others [40]. We initialized the maximum fitness cost of each epitope in the beginning of the simulation by drawing it from a uniform distribution, $c_{i}^{*}∼U\;[0,\;c_{\max}]$. We assumed that neutralization via antibodies is the primary driver of selection in chronic infection, and that virus is saturated with antibodies. In this scenario, the fitness loss of a virus variant upon antibody recognition is driven by the most potent antibody that binds it, $c_{j}^{imm}=max\left(c_{ij}\delta_{ij}\right)$. The distribution of fitness costs associated with the epitopes defines the fitness landscape, which influences the evolutionary trajectory of the virus population.

Purifying selection conserves sites in the HIV-1 genome where mutations would be deleterious to the virus. We assume that mutations at invariant sites (where no variability is observed across sequences from different time points) are inherently deleterious, and incur a multiplicative fitness cost, $c_{j}^{inv}=1-{\left(1-\psi \right)}^{k}$, where *ψ* is the reduction in fitness per mutation, and *k* is the number of mutations. At the beginning of each simulation, we randomly distribute *Lp*_*inv*_ invariant sites across the sequence, where *p*_*inv*_ is the proportion of invariant sites. Note that the positions of the invariant sites vary between simulations, and may occur in both non-epitope and epitope regions. Following Ganusov et al. [41], we define the relative fitness of variant *j* as $f_{j}=\left(1-c_{j}^{imm}\right)\left(1-c_{j}^{inv}\right)=\left(1-max\left(c_{ij}\delta_{ij}\right)\right){\left(1-\psi \right)}^{k}$.

### Selection of parameter values

We considered a pool of *N*_*C*_ = 15000 infected cells, which is in line with the mean effective population size of HIV-1 in chronic infection [18]. HIV-1 has a large burst size of approximately 50,000 [42], but only a small fraction of one in 1000 to 10,000 virions appear to be infectious [43–45]. Each productively infected cell therefore produces between 5 and 50 infectious virions. For consistency with the sequence evolution models developed by Vijay et al. and Balagam et al., we set the number of progeny virions produced by each productively infected cell to *P* = 5 [17, 18]. Following again Balagam et al., we set the generation time to 1.2 days [18], and the substitution rate to the mean of the mutation rates estimated by Mansky et al., *μ* = 3.5 × 10 − 5 [46]. We set the proportion of invariant sites to 50%, corresponding to the mean proportion of invariant sites estimated from the HIV-1 sequence alignments of patients described in Bunnik et al. and Karlsson et al. [9, 22]. We ran the simulations for 3000 generations, corresponding to 10 years.

To maintain the ratio of productively infected to latent cells predicted by the homeostatic proliferation model of latency introduced by Kim et al. and Rong et al., we set the size of the latent reservoir to *N*_*L*_ = 100 [12, 13]. Assuming a total body load of 2 × 10^8^ productively infected cells [47] and between 2.2 × 10^5^ to 1.6 × 10^7^ latent cells with replication competent DNA [48], there are between 0.0011 to 0.08 latent cells for every productively infected cell. In our simulations with 15,000 productively infected cells, this corresponds to a latent reservoir size *N*_*L*_ of 16 to 1200 cells; our chosen latent reservoir size is close to the geometric mean of this range.

We chose a conservative estimate of 44 months for the half life of the latent reservoir [33, 36], which we used to calibrate the probability *η* of an infected cell becoming latent upon infection such that the size of the latent reservoir was maintained. Archin et al. recently estimated that the composite parameter *βη* is on the order of 10^−14^ [15], where *β* is the mass-action infection rate constant in *ml* ^−1^ *day* ^−1^. The latter has been estimated to be approximately *β* = 1.5 × 10^−8^ [49, 50]. Our value of *η* = 3.5 × 10^−6^ is therefore in line with these estimates. We set the death rate of latent cells to *d*_*L*_ = 0.004 per generation, corresponding to the estimated death rate of memory cells, which make up the bulk of the latent reservoir [13, 14]. Following Rong et al., we varied the activation rate from *a*_*L*_ ∈ (0.001,0.01) per generation so that between 0.1 and 1 latent cells were activated each generation [13, 14].

Barr et al. estimated that the minimum efficacy of three early neutralizing antibodies at blocking *de novo* infections ranged from 19.6% to 35.2% [10]. To account for the observation of Richman et al. [7] that some antibodies isolated from patient serum show no neutralization activity, we allowed the maximum fitness cost, $c_{i}^{*}$, due to recognition of a viral epitope to range from 0 to 0.40, with a mean of 0.2.

The remaining parameters associated with the immune response are not well known. For our default parameter setting, we assumed that there are ten epitopes in the approximately 700 nucleotide region of envelope that we simulate. In subsequent sensitivity analyses, we varied the number of epitopes between 0 and 15. While it is not known how many epitopes there are on the envelope, analysis of escape mutations to SIV in rhesus monkeys suggests that there are several [51]. We assumed that an antibody is created against an epitope variant once it reaches 1% frequency in the plasma. In sensitivity analyses, we varied the antigen frequency required for stimulating a new antibody response between 0.1% and 10%. Because patient serum does not neutralize contemporaneous virus but rather virus that circulated in the plasma at least 3 months earlier [7], we assumed that it takes 90 generations for a newly introduced immune response to reach its full potency (i.e. impose maximal fitness cost to the virus variant it recognizes, with fitness cost increasing linearly from zero over the 90 generations). The default values of the model parameters are summarized in Table 1.

**Table 1:** Parameters used in our model.

Parameter	Value	Description
*N*_*C*_	15000	Number of cells in the replicating population [18]
*N*_*L*_	100	Number of cells in the latent reservoir [13]
*τ*	1.2	Duration of each generation [days] [18]
*L*	669	Length of simulated DNA sequences [nucleotides] [9, 21, 22]
*μ*	3.5 × 10^−5^	Mutation rate per site per generation [46]
*N*_*R*_	150	Mean number of recombination events per generation [26–28]
*P*	5	Number of progeny virus produced by each infected cell [17, 42]
*η*	3.5 × 10^−6^	Probability of infected cell becoming latent, calibrated to maintain size of latent reservoir, assuming 44 month half-life [35, 36]
*a*_*L*_	0.001 − 0.01	Activation rate of latent cells [13, 14]
*δ*	0.004	Death rate of latent cells [13, 14]
*N*_*E*_	10 (0–15)	Number of epitopes [51]
*L*_*E*_	20	Length of epitopes, based on non-synonymous sites [nucleotides] [38]
$c_{i}^{*}$	*U*[0,0.4]	Maximum fitness cost of epitope *i* upon recognition [10]
*p*_*inv*_	0.5	Proportion of invariant sites [9, 21, 22]
*ψ*	0.9	Fitness cost per mutation on invariant site
*f*	0.01 (0.001- 0.1)	Proportion new viral antigen must reach to initiate immune response against corresponding epitope
*d*	90	Number of generations for a new immune response to reach full potency

To investigate the robustness of our results, we also considered alternative parameter values proposed in the literature. Recent estimates of the basic reproductive number of HIV-1 during primary infection suggest that an infectious cell generates at least eight new infected cells at the start of infection when target cells are not limiting. Thus, each infected cell produces at least *P* = 8 infectious progeny virions [52]. Following Pearson et al., we set the number of progeny virions to *P* = 10 to account for viral clearance [53]. We set the generation time of HIV-1 *in vivo* to two days, estimated from the decay dynamics of productively infected cells [54], and the substitution rate to the reverse transcriptase nucleotide substitution rate of approximately 2.2 × 10^−5^ [46, 55]. We further assumed that 10% of sites in the envelope region of interest are invariant, corresponding to the minimum proportion of invariant sites in the patient data. Because the mechanism of recombination is central to our investigation, we also varied the number of recombination events per generation between 0.1*N*_*R*_, and 10*N*_*R*_.

### Sequence divergence and diversity

Estimating sequence divergence and diversity allows us to quantify viral evolution. Divergence is a measure of how far viral genomes have evolved from the founder strain, whereas diversity is a measure of the genomic variation in the viral population at any given time. Every 30 generations, we calculated the divergence and diversity of the HIV-1 sequences in the entire populations of productively infected and latent cells, recording the mean, median, and 5% and 95% quantiles. Furthermore, we randomly sampled 100 productively infected cells and 100 cells from the latent reservoir every 300 generations, storing the HIV-1 sequences for later phylogenetic analysis. Using *d*(*i*, *j*) to denote the mean number of differences per position between sequences *i* and *j*, divergence and diversity are defined as follows for a collection of *k* sequences:

$$\begin{array}{c} divergence=\frac{1}{k}\sum_{i=1}^{k}d_{i,founder} \end{array}$$

$$\begin{array}{c} diversity=\frac{\sum_{i=1}^{k-1}\sum_{j=1+1}^{k}d\left(i,j\right)}{\frac{1}{2}k\left(k-1\right)}. \end{array}$$

### The effect of simulated population size on survival of latent genomic fragments

Stochastic effects dominate at small population sizes—the smaller the population, the larger the probability that stochastic fluctuations lead to extinction. We therefore investigated how the size of the simulated population affects the ability of latent genomic fragments to survive in patient plasma, assuming that recombination is necessary for survival. To this end we determined the expected number of dual infections, a precursor for recombination, involving at least one latent form when the sizes of the replicating and latent populations were increased ten-fold. We ran 100 simulations, tracking the progeny of the activated latent cells for ten generations, introduced into a replicating population of size *n*_*r*_ at generation zero. The relative fitness of each latent virus was set to 0.5, while the relative fitness of each non-latent virus was drawn from a uniform distribution between 0.5 and 1. As in our full model, each generation of the simulation consisted of every infected cell producing five progeny virions, and downsampling the virus population to infect the next generation of *n*_*r*_ uninfected cells. The expected number of dual infections involving at least one latent virus was then estimated for each simulation run.

### Patient data

Simulation results were compared to HIV-1 DNA sequence data from 16 untreated or unsuccessfully treated patients [9, 21, 22]. All patients were infected with HIV-1 subtype B. The patients were followed longitudinally from seroconversion with 2–22 sequences sampled at intervals of 1–67 months (mean 13 months). The sequences were derived from the envelope, and were between 532–948 nucleotides long (mean 683 nucleotides). For each patient, we calculated the mean divergence and diversity at each time point the same way as for the simulated data. Note that in [21], sequence divergence and diversity were estimated from mean pair-wise distances determined using either a two-parameter Kimura model or a general time-reversible model with site-to-site variation in substitution rates, while here only simple pairwise Hamming distances were used. We considered the unsuccessfully treated patients as effectively untreated, because their viremia was not suppressed, and their divergence and diversity trends were similar to those of the untreated patients. We also estimated the proportion of invariant sites across all time points for each patient using PhyML [56]. The mean proportion of invariant sites across all patients was 39%. The lowest proportion of invariant sites was 13% for Patient 9 in the Shankarappa cohort, while the mean was greater than 50% for both the Amsterdam and Karlsson patients.

## Results

### Simulated HIV-1 genetic trends emulate real patient data

**Fig 2:**
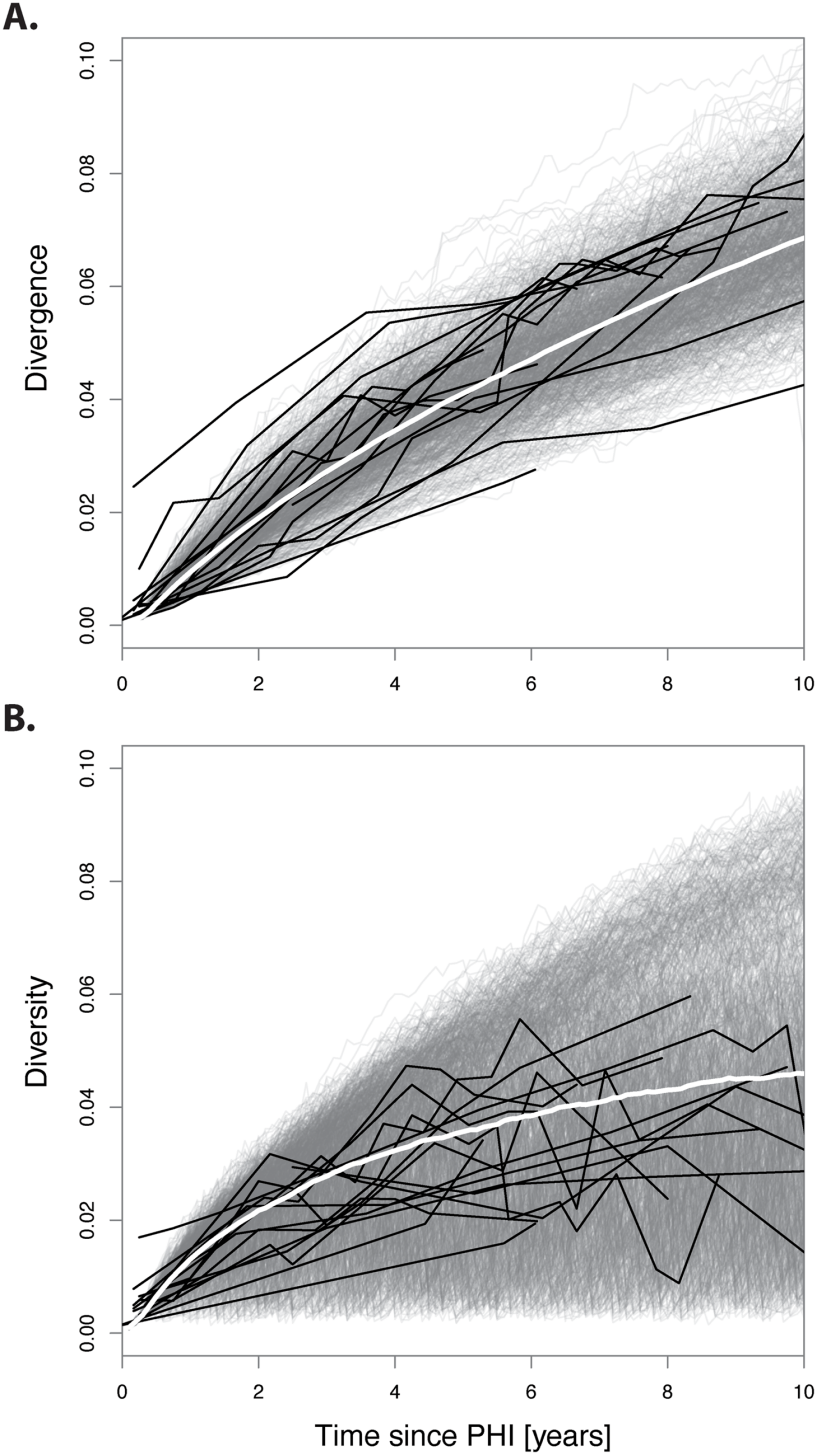
The divergence and diversity of simulated sequences capture evolutionary trends in clinical data. Grey lines correspond to the divergence (A) or diversity (B) of the sequences in each simulation, and white lines show mean of simulations. Black lines show real patient data. Note that divergence and diversity were calculated every 30 generations for 15,000 sequences in each of the 1000 simulations.

**Fig 3:**
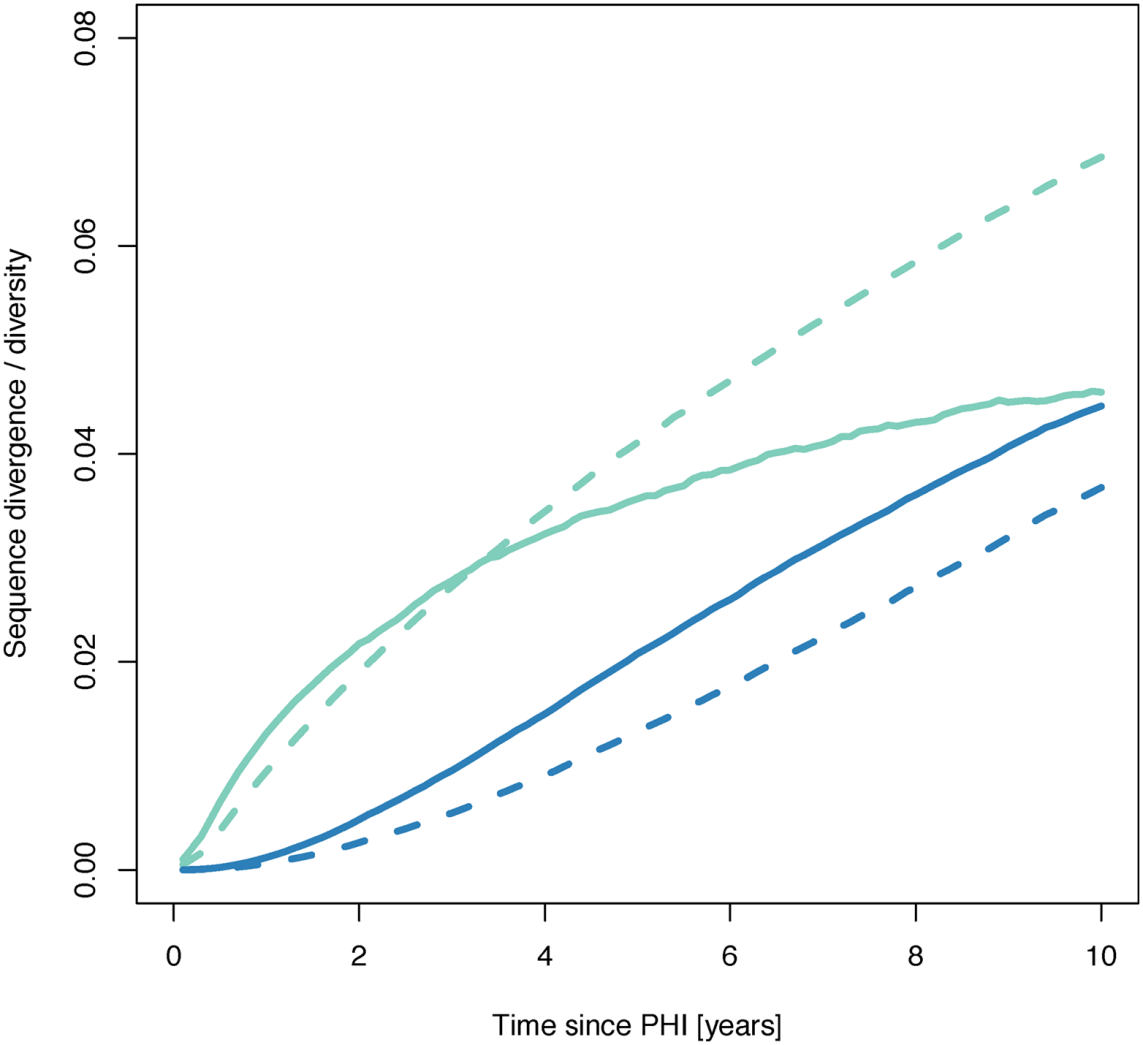
The divergence and diversity of simulated sequences in the latent reservoir initially increase much more slowly than in productively infected cells. After approximately 2 years post-PHI, diversity grows linearly in the latent reservoir (blue solid line) while it starts to saturate in plasma (green solid line). Divergence in the latent reservoir (blue dashed line) grows at approximately the same rate as in the plasma (green dashed line) 6 years post-PHI.

To be sure that our model invokes realistic trends of HIV-1 genetic evolution, we compared our simulated patient HIV-1 populations generated by simulations incorporating recombination to HIV-1 DNA sequence data from 16 untreated or unsuccessfully treated patients [9, 21, 22]. Reassuringly, the empirical HIV-1 diversity and divergence trends over time were qualitatively and quantitatively similar to our simulated patient HIV-1 populations (Fig 2). Divergence increased linearly over time to a mean of 0.06 substitutions per site at 10 years post-primary HIV-1 infection (post-PHI), while diversity initially increased rapidly, and then saturated to approximately 0.05 substitutions per site at 10 years post-PHI. As seen in S1 Table in the supplement, there was no significant trend between the activation rate and mean sequence diversity (*p* = 0.67, *cor* = 0.15; Pearson’s product-moment correlation). Increasing the activation rate slightly decreased sequence divergence at the end of the simulations (*p* = 0.04, *cor* = −0.65; Pearson’s product-moment correlation). Unsurprisingly, both mean sequence divergence and diversity increased in the latent reservoir at a much slower initial rate than in productively infected cells (Fig 3). After approximately 6 years post-PHI, divergence in the latent reservoir proceeded at nearly the same rate as in productively infected cells. Because diversity in the latent reservoir grew linearly but started to saturate in plasma, by 10 years post-PHI diversity in the latent reservoir almost reached that in productively infected cells.

### Fitness landscape influences tree shape and genetic trends

**Fig 4:**
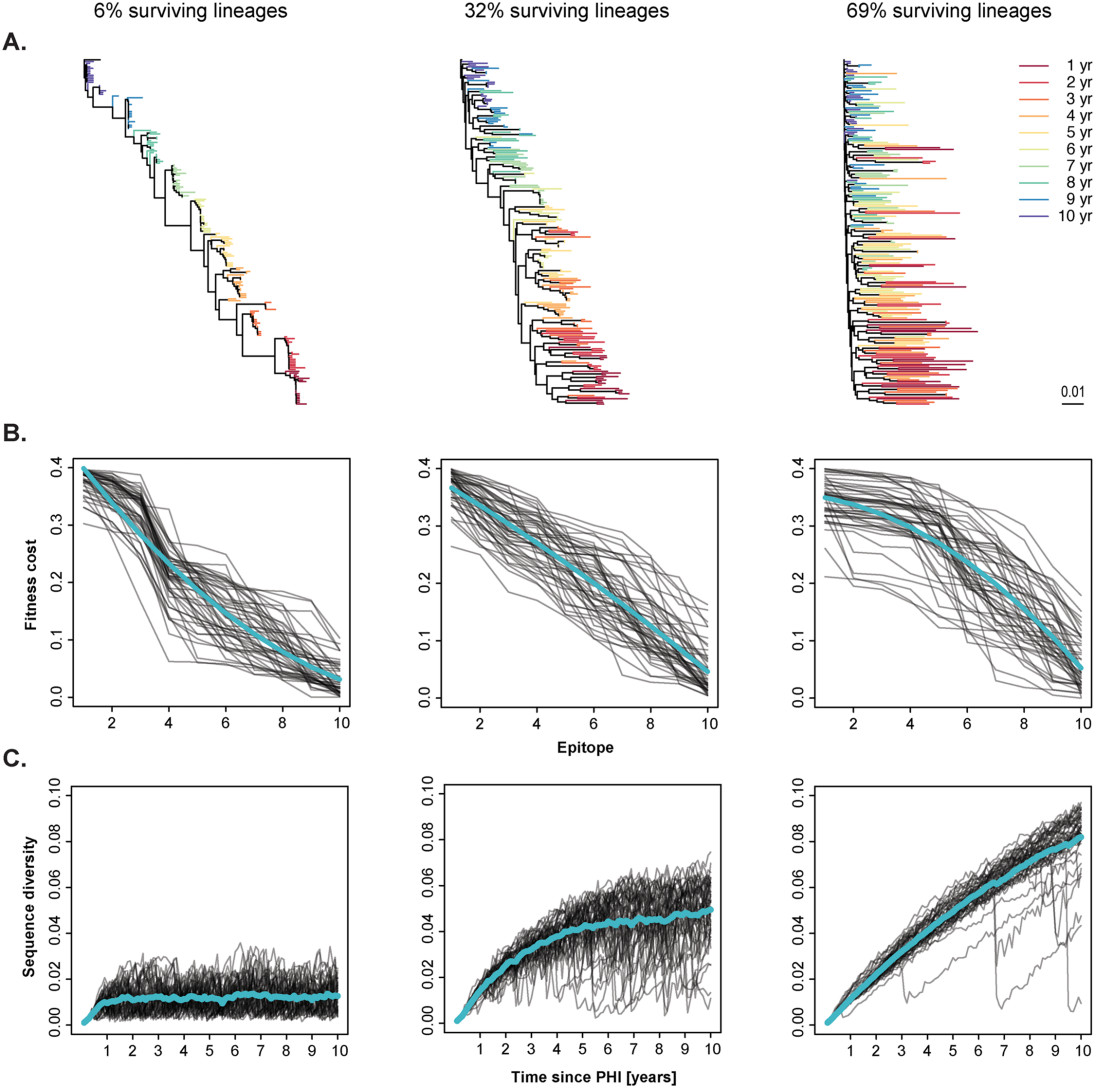
Tree shape and diversity are influenced by the fitness landscape. We ordered the simulations based on the average proportion of phylogenetic lineages surviving between samplings, and formed three groups consisting of 50 simulations each, with low (mean 6%), intermediate (mean 32%), and high (mean 69%) survival of lineages, respectively. (A) Typical trees of each group. Branch lengths are according to the indicated scale. Color indicates sampling time. (B) Individual fitness landscapes (grey lines) and average profile (quadratic fit, turquoise lines) of each group. (C) Individual diversity curves (grey lines) and average trends (turquoise lines).

Reconstructed phylogenies of the simulated patient HIV-1 populations, with 20 sequences sampled every 12 months, showed similar topological structures as patient HIV-1 populations. While the serially sampled HIV-1 phylogenies ranged from star- to ladder-like structures, they generally displayed a clear time trend (Fig 4). Both in the simulated and clinical data, the fraction of surviving phylogenetic lineages between samplings ranged from 0.1 to 1.0 (S1 Fig). Low survival of lineages corresponds to a ladder-like phylogeny while high survival corresponds to a star-like phylogeny.

The tree shapes were largely explained by the fitness landscape, where convex profiles lead to more ladder-like trees, and concave profiles lead to bushier, star-like, trees. Intermediate fitness landscapes (nearly linear profiles) generated trees more typically observed in HIV-1 infected patients. Importantly, these intermediate landscapes also showed typical patient diversity trends (Fig 4). Interestingly, in intermediate and concave profiles we sometimes observed dramatic selective sweeps where the diversity dropped drastically and then recovered. The fitness landscape also influenced the number of latent origins (latent virus of different ages reaching at least 1% frequency in plasma virus population). The mean (s.d.) number of different latent origins was 0.16 (0.42) for convex landscapes, 0.42 (0.76) for nearly linear landscapes, and 7.6 (7.5) for concave ones. Thus, our simulations suggest that the fitness landscape influences general tree shape, diversity trends, and the survival of latent forms.

The shape of the landscape is defined by the maximum fitness costs exerted by immune responses against different viral epitopes. The concave landscapes have a higher number of different immune responses exerting similar high fitness costs on the virus than convex or nearly linear landscapes. Escaping the immune response exerting the maximum fitness cost only marginally increases fitness if there are simultaneous immune responses against other epitopes that also impose high fitness costs. Therefore, a more concave fitness landscape exerts a stronger selection pressure on the virus, reducing both the mean and variance of the relative fitness in the virus population. The survival of less adapted latent forms in plasma therefore increases, while the reduction in fitness differences between virus variants in productively infected cells increases diversity.

### Latency increases sequence diversity

**Fig 5:**
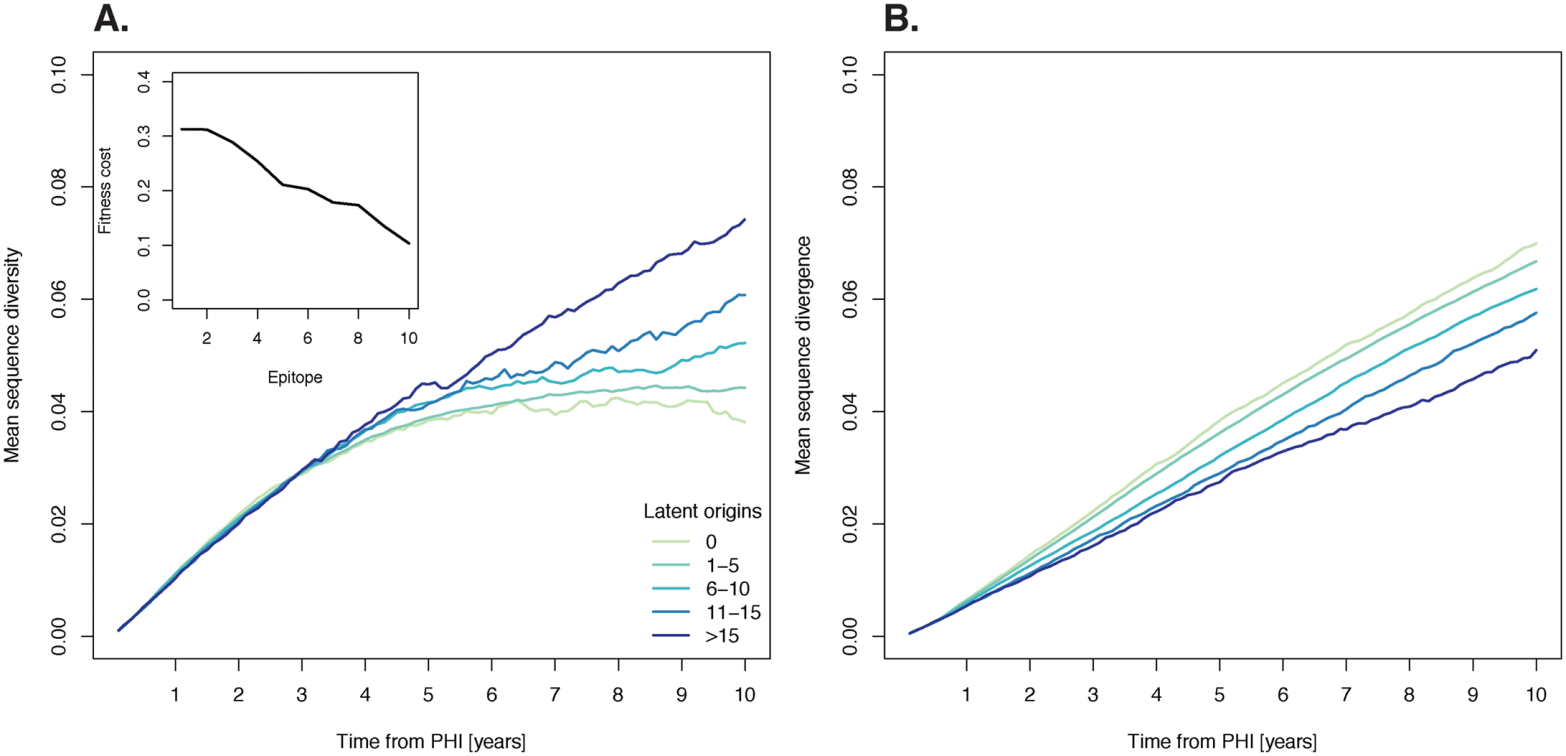
Survival of different latent genomic fragments increases sequence diversity. We ran 1000 simulations with the intermediate fitness landscape shown in the insert, and categorized the results based on the number of latent genomic fragments from different origins at 1% or greater frequency at 10 years post-PHI. (A) As the number of latent origins increases, so does mean sequence diversity. (B) As the number of latent origins increases, mean sequence divergence decreases.

Fig 5 shows that the more genomic fragments with different latent origins persist in the plasma, the higher the sequence diversity (panel A). Compared to simulations with no surviving latent origins, sequence diversity became significantly higher after 9.5 years post-PHI in simulations where there were between 1 and 5 latent origins (*p* < 0.05, Wilcoxon rank-sum test), while in simulations where there were more than 5 latent origins, sequence diversity became significantly higher much earlier, after approximately 5 years post-PHI (*p* < 0.05, Wilcoxon rank-sum test). At 10 years post-PHI, mean sequence diversity was 0.038 substitutions/site when no latent genomic fragments survived, increasing by 16%, when there were between 1 and 5 latent origins, and by 95% when there were more than 15 latent origins. The propagation of genomic fragments that have accumulated fewer mutations than contemporaneous variants should reduce average sequence divergence in the plasma. As expected, when the number of surviving genomic fragments of different latent origins increased, mean sequence divergence decreased (Fig 5, panel B).

Our model predicts that multiple introductions of latent forms into plasma are necessary for increased diversity; simulations with only one surviving latent origin did not have significantly higher diversity than those with no latent forms in plasma. Because sequence diversity increased almost linearly for concave fitness landscapes, the introduction of older virus forms into plasma did not further increase diversity (S2 Fig). Furthermore, because the survival of multiple latent origins was rare for convex fitness landscapes, it was not possible to determine whether latency could have had an effect on diversity under such conditions. However, the phylogenies and diversity trends generated by convex or concave fitness landscapes did not resemble those observed in typical patients.

### Recombination facilitates survival of latent HIV-1 genomic fragments

**Fig 6:**
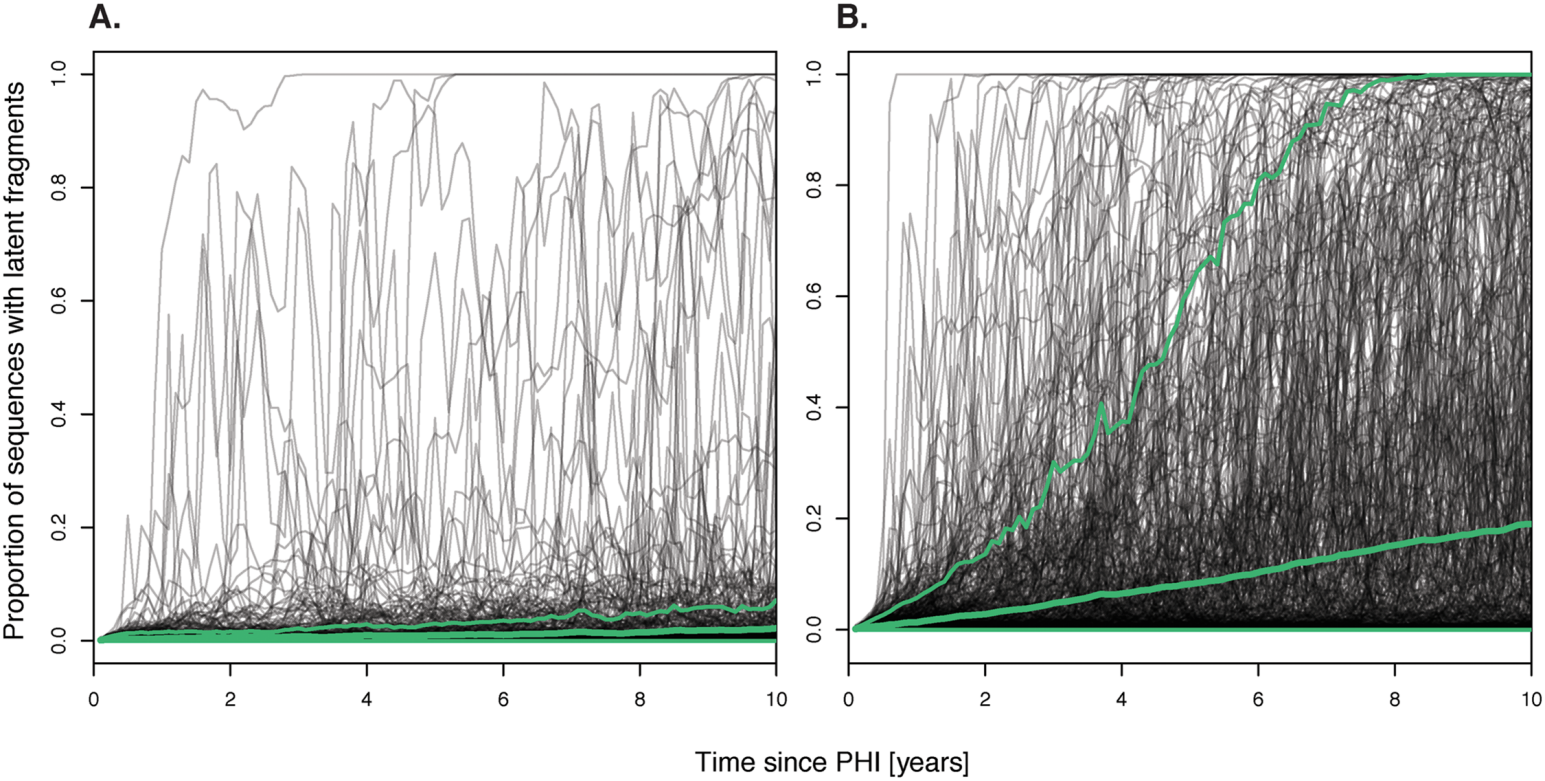
The effect of recombination on survival of activated latent HIV in the plasma population. A) Simulations without recombination. B) Simulations with recombination. Grey lines show the proportion of virus with latent genomic fragments in the productively infected cell population of individual simulations, where the bold green line is the mean proportion and the thin green lines outline the 95% confidence envelope. Comparing panels A and B, clearly shows that recombination facilitates survival of latent forms.

Fig 6 shows the effect of recombination on the proportion of virus with latent genomic fragments in productively infected cells in 2000 simulations of untreated patients. Our simulation results suggest that recombination facilitates the survival of latent genomic fragments. In simulations with recombination, 27% of the HIV-1 replicating plasma populations reached a mean of ≥ 10% virus with latent genomic fragments between 5–10 years post-PHI, while only 2.0% reached ≥ 10% latent forms without recombination. Thus, recombination increased the survival of latent genomic fragments in productively infected cells approximately 13-fold.

The proportion of virus with latent genomic fragments was higher when we allowed for recombination than when we ignored it. This distinction is clear from even the first sample time, after 30 generations (*p* < 0.05, Wilcoxon rank-sum test). Furthermore, the rate of increase of virus with latent genomic fragments over time in the replicating population was approximately 9 times faster when recombination was involved Fig 6. In most simulations without recombination, virus with latent fragments immediately disappeared from productively infected cells upon introduction, while in some simulations, sharp peaks were observed where the proportion of latent forms increased rapidly to a high fraction but then fell quickly and disappeared completely. In simulations with recombination, the proportion of virus with latent genomic fragments often increased gradually, and stabilized both at intermediate values and the extremes.

### Latent HIV display many different recombination patterns

**Fig 7:**
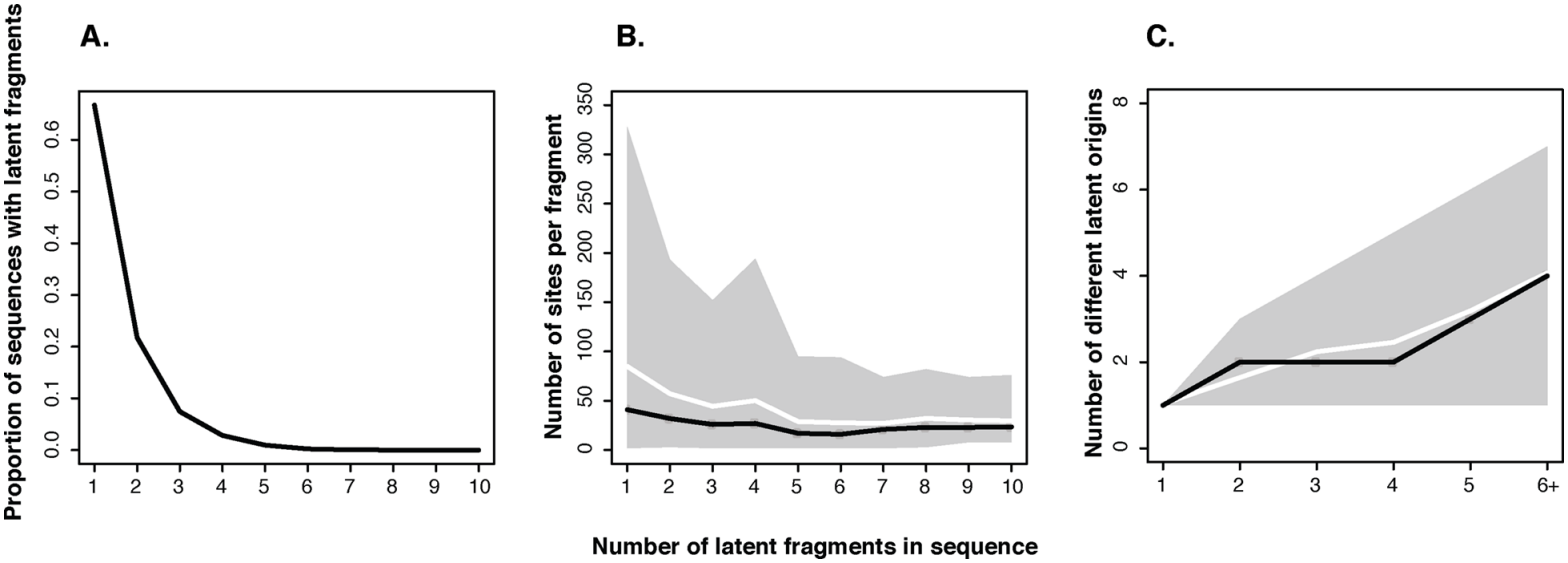
Latent genomic fragment patterns. (A) The proportion of sequences with latent fragments declines rapidly as the number of latent fragments per sequence increases. (B) The number of latent sites in recombinants decreases as more latent fragments are introduced. (C) As more latent fragments exist in a sequence, they have different origins in time. The grey envelopes indicate 95% of simulation results, white lines indicate the mean, and the black lines the median trends.

Most recombinant HIV-1 that include a fragment from a latent virus underwent relatively few recombination events; 96% had fewer than 5 latent fragments (Fig 7A). However, only 0.7% of sequences with latent sites at 10 years post-PHI had not acquired genomic fragments from non-latent contemporaneous virus through recombination. The most common latent recombinant observed in our simulations had one latent fragment, with a mean (s.d.) of 85 (123) sites (Fig 7A). When such a recombinant proliferates further, additional recombination with contemporaneous virus removes sites from the latent fragment. Overall, the mean (s.d.) number of latent sites at 10 years post-PHI was 99 (121), while the mean (s.d.) fragment length was 66 sites (Fig 7B).

When the number of latent fragments in a viral sequence increased, the fragment length decreased (Fig 7B). Interestingly, viruses that had more latent fragments also had more latent origins (Fig 7C). Hence, the most common forms of latent recombinants had few breakpoints, and multiple breakpoints frequently involved latent virus with different origins in time. Increasing the recombination rate increased the survival of virus with latent genomic fragments (S2 Table). As expected, the number of latent fragments per sequence also increased, while the fragment length decreased.

### Many phylogenetic lineages over time implies many different latent origins

**Fig 8:**
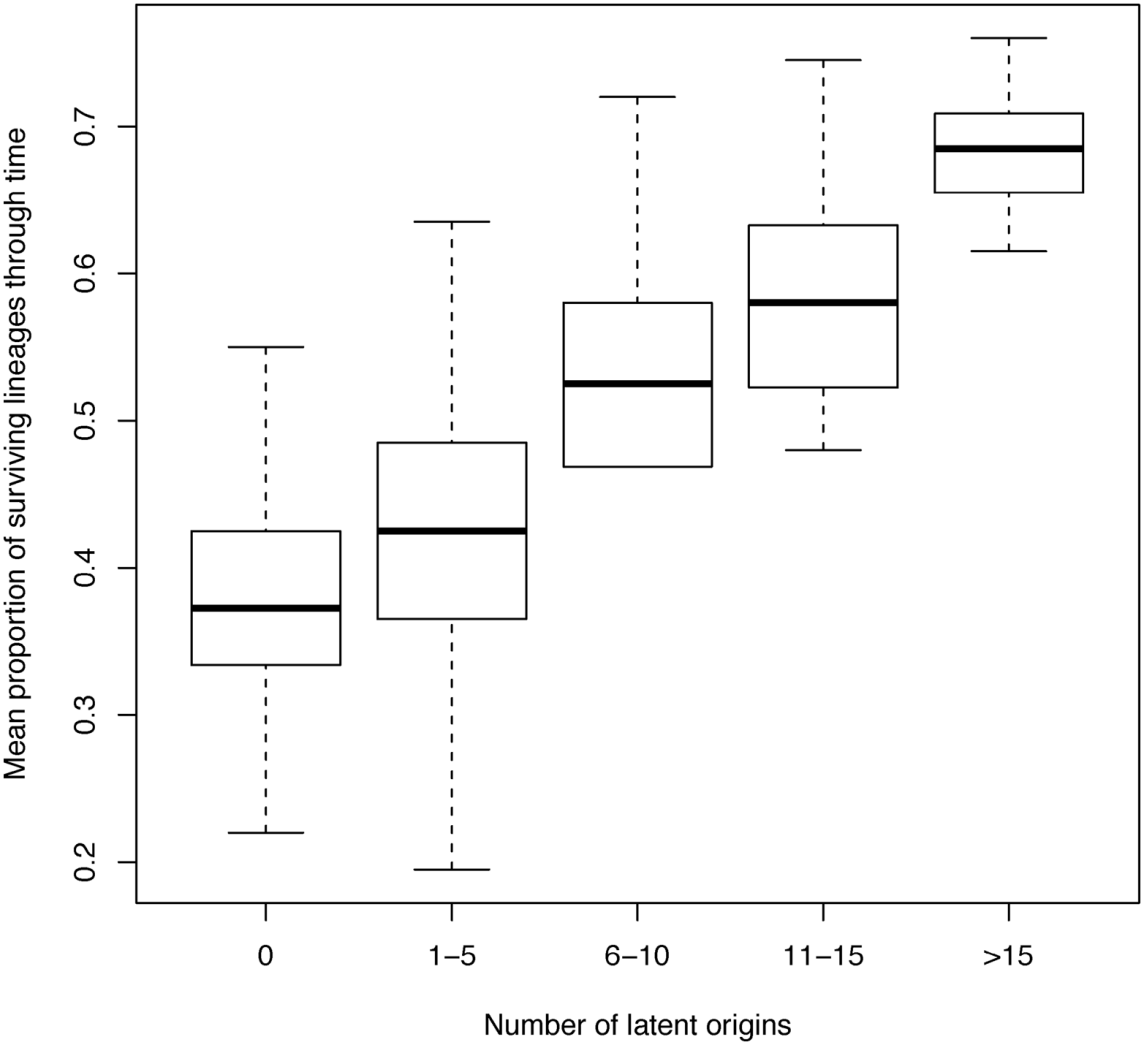
Survival of lineages through time increases with number of latent origins. The boxplots show the distributions of the mean proportion of lineages through time (averaged from 0 to 10 years post-PHI for the phylogeny generated from each simulation) when the number of latent genomic fragments from different origins increases. The survival of lineages increases almost linearly with the number of latent origins.

Higher proportions of surviving lineages through time imply higher numbers of virus with genomic fragments of different latent origins that have long branches reaching far back into the phylogeny (Fig 8). When the prevalence of latent genomic fragments in productively infected cells was high, they were typically of different latency ages, i.e., deposited and activated at different times (S3 Fig). These observations are supported by the noted increase in both the proportion of virus with latent genomic fragments and the observed number of different latent origins in productively infected cells when the activation rate was increased (S4 Fig).

### Recent latent forms are more common in plasma even when old latent forms are overrepresented in the reservoir

**Fig 9:**
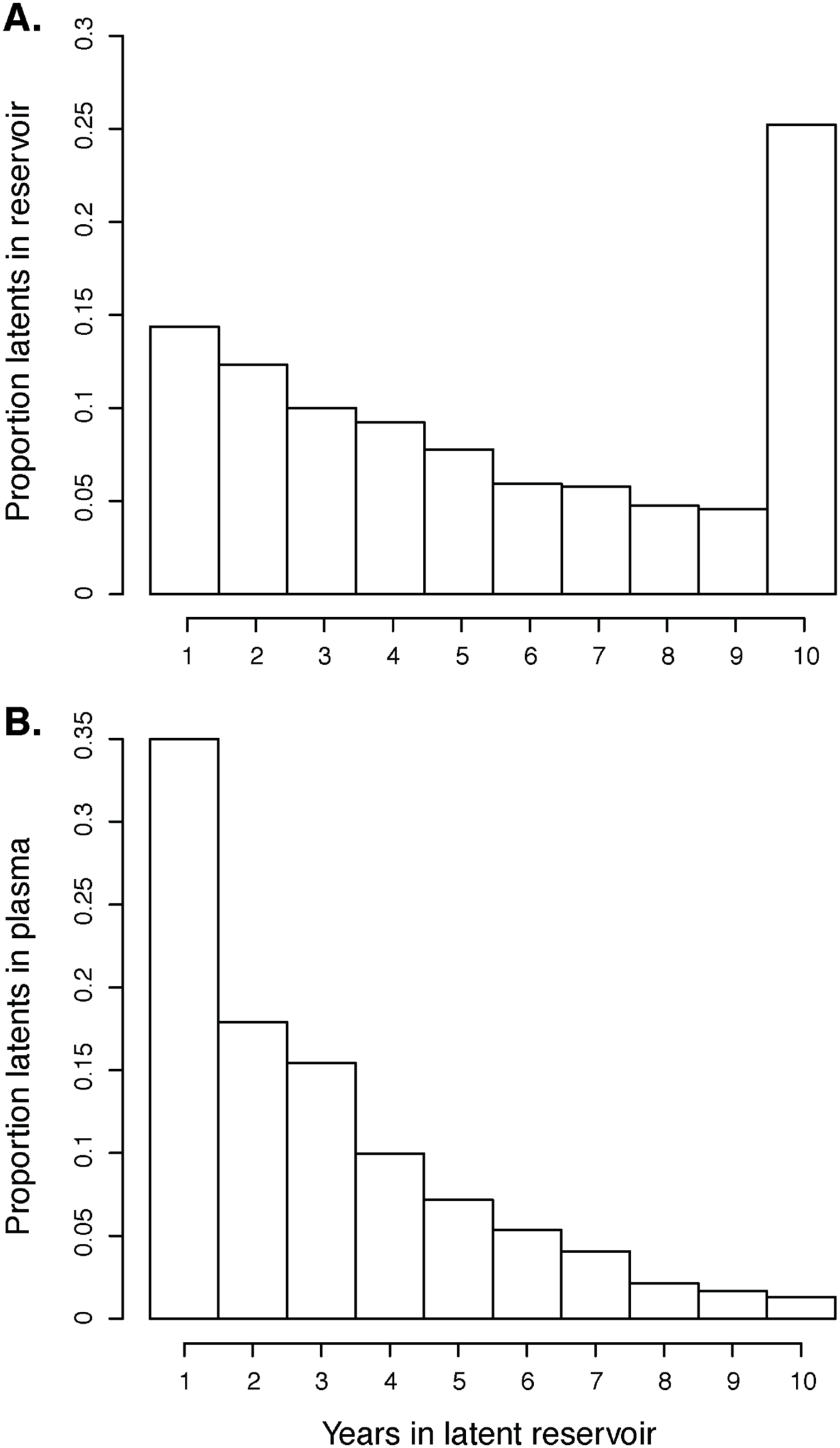
Distribution of latency periods in the latent reservoir and productively infected cells. (A) Distribution of time that cells have spent in the latent reservoir. (B) Distribution of the time that latent genomic fragments in plasma have spent in the latent reservoir.

When a person becomes infected with HIV-1 the initial spike in viral load may deposit many similar copies of the initial virus in the reservoir. We modeled this by initially filling the reservoir with the infecting strain. Thereafter, a relatively slow deposit rate and proliferation rate refill and maintain a diversifying latent virus population. Interestingly, at 10 years post-PHI, our simulations predict that about 27% of the reservoir still consists of virus deposited in the first year of infection (Fig 9A). Conversely, the latent genomic fragments in the plasma population originated mostly from more recently deposited and activated virus (Fig 9B). Overall, the age structure among latent genomic HIV-1 fragments in the plasma of an untreated patient followed that of the latent reservoir with the exception of the initially deposited virus, which is strongly selected against upon activation by the refined immune response.

### Survival of latent genomic fragments under recombination is robust to perturbations of the model parameters

The evolutionary trends of the virus populations simulated under the alternative model parameters (*P* = 10, *μ* = 2.2 × 10^−5^, *inv* = 0.1, *τ* = 2 days) were qualitatively and quantitatively similar to those seen previously under the default parameters. The mean sequence divergence and diversity of 100 simulations with the alternative model parameters are shown in (S5 Fig). We found that the prevalence of virus with latent genomic fragments in productively infected cells was much higher in simulations with recombination than without (S6 Fig). Our simulation results are therefore robust to the choice of parameters defining the infection and replication dynamics.

The sensitivity of the model to the immune system parameters, i.e., the antigen frequency necessary to elicit a new immune response, and the number of epitopes accessible to the immune system, are described in (S3 and S4 Tables), respectively. Fifty simulation runs were performed for each parameter value. When the antigen frequency required for stimulating a new immune response was increased from 0.1% to 10%, the survival of virus with latent fragments and sequence diversity decreased (*p* = 0.025, cor = -0.67; *p* = 0.013, cor = -0.71, respectively; Pearson’s product-moment correlation), while sequence divergence increased (*p* = 0.003, cor = 0.79, Pearson’s product-moment correlation).

When the immune response to a new escape mutant is delayed, the frequency of virus variants carrying the advantageous escape mutation increases. The increase in the number of virus variants that have evolved away from the founder sequence increases divergence but the simultaneous convergence towards the new escape variant reduces diversity. Because the mean relative fitness of virus in productively infected cells increases, the survival probability of virus from activated latent cells decreases. The survival of virus with latent genomic fragments was low in all simulations without recombination.

On the other hand, when the number of epitopes was increased, sequence diversity at 10 years post-PHI initially decreased from one epitope to 4 epitopes and then increased from 4 to 15 epitopes (S4 Table). Sequence divergence initially increased from one to 4 epitopes, and then slightly decreased as the number of epitopes was increased. The survival of virus with latent genomic fragments was more stochastic, but generally displayed a similar trend. Virus with latent genomic fragments rarely survived in simulations without recombination. Therefore, our fundamental model result suggesting that recombination is an essential mechanism facilitating the survival of latent genomic fragments is robust to large perturbations in the largely unknown parameters describing the immune response.

When the number of epitopes was zero, no immune pressure was exerted on the virus. This resulted in high diversity (0.081 subst/site) because all virus variants without mutations at invariant sites had equal probability of surviving, and low divergence (0.047 subst/site), because rapid accumulation of escape mutations was not selected for. Virus with latent genomic fragments survived in all simulations, because earlier virus forms were not at a fitness disadvantage compared to more recent variants.

When the number of epitopes was increased from 1 to 4, it became increasingly more difficult but not impossible for virus to escape the immune response. When two epitopes had high fitness costs associated with them, it took longer for any virus variant in the population to simultaneously escape both responses, and the significant fitness gain and proliferation due to escape resulted in decreased diversity (S7 Fig). Such genetic sweeps were more dramatic when three epitopes had high fitness costs. When four epitopes had high fitness costs, escape at all epitopes to gain fitness was nearly impossible, and happened in only a small fraction of the simulations. Therefore, more concave fitness landscapes with four or more epitopes with high fitness costs imposed selection pressure resulting in high diversity (S7 Fig). The probability that a randomly drawn fitness landscapes had 2 or 3 epitopes with high fitness costs was highest when the total number of epitopes was 4 or 5. When the number of epitopes was increased from 6 to 15, the probability that each fitness landscape had 4 or more epitopes of high fitness costs also increased.

### Small sample size may underestimate the survival of latent genomic fragments

Surviving in the replicating population long enough to recombine with a replicating virus forms the bottleneck that largely determines the long-term fate of a latent lineage. When one latent form was introduced into a population of 15,000 cells at generation *t* = *t*_0_, corresponding to the expected number of cells activated each generation from the latent reservoir of 100 cells at the highest activation rate *α* = 0.01, the expected number of dual infections involving at least one latent virus was 0.1 over 10 generations. However, when 10 latent forms were introduced to a population of 150,000 cells, i.e., at the same proportion but in a ten-fold population, the total expected number of dual infections was 0.94. Thus, when the simulated population size was increased ten-fold, the probability that at least one latent form ended up in a dually infected cell before disappearing from the replicating population increased nearly ten-fold. The long-term behavior in simulations where virus with latent genomic fragments proliferates is expected to be robust to stochastic effects due to the increased number of such viruses, however, the variance observed in the survival of latent virus forms between simulations is overestimated in a smaller population due to early stochastic extinction. The proportion of simulations with persistence of virus with latent genomic fragments is therefore likely an underestimate.

## Discussion

We developed a within-host HIV-1 evolution model that includes point mutation, recombination, immune (positive) selection, negative selection, and latency. This model quantitatively captures previously observed viral sequence divergence and diversity trends and mechanistically explains these patterns, allowing us to model realistic within-host natural evolution of HIV-1. We used this model to investigate when and how latent forms of HIV-1 can survive in productively infected cells despite not being well adapted to the immune response, therefore having reduced relative fitness. Our simulation results suggest that recombination is a key mechanism facilitating the survival of virus forms with latent genomic fragments. We have previously shown that the majority of phylogenetic lineages in HIV-1 populations of untreated patients display a statistically defined signal of latency [1]. The results in this paper suggest that such lineages likely survived because of recombination. Our model further predicts that the survival of latent genomic fragments in plasma depends on the fitness landscape induced by the immune response.

By comparing simulations where the surviving genomic fragments originated from different numbers of latent ancestors, we found that latency reduced mean sequence divergence, but increased mean sequence diversity for reasonable fitness landscapes. This effect may explain how HIV-1 can keep a high adaptation potential without mutating too far away from the infecting strain. High adaptation potential is useful for escaping immune and antiviral drug pressures, while less divergent sequences closer to the infecting form are arguably more fit to infect new hosts [2].

The dynamics of our simulated immune response depend on the number of epitopes and the antigen frequency triggering a new immune response, neither of which are well known. To test the sensitivity of our model, we varied these parameters to the extremes where measures of sequence divergence and diversity became unrealistic. Importantly, the trend of much higher survival of virus with latent genomic fragments in simulations with recombination than in simulations without recombination was robust for the full range of immune system parameters. This suggests that our main result is not sensitive to the exact details of how the immune response was implemented in our model.

While *in vivo* HIV-1 viral populations are typically very large, computational considerations limited us to follow instead the relatively small population of 15,000 productively infected cells, which is in line with recent estimates of the effective population size in real HIV-1 populations [18]. The variability in the survival of virus with latent genomic fragments observed in our simulations is likely a result of stochastic fluctuations when latent virus first enters the replicating population. The proportion of simulations in which latent virus forms survive is therefore likely to be an underestimate. However, we expect the mean dynamics of virus with latent genomic fragments to be robust to scaling issues after the initial bottleneck.

Previous studies have suggested that escape mutations have a fitness cost [10], and thus virus closer to the infecting strain may have higher replicative fitness due to having accumulated fewer escape mutations to evade immune surveillance. One of the limitations of our model is that we do not consider fitness costs associated with immune escape. However, compensatory mutations may arise in other parts of the genome, and at least partially restore fitness [11]. Furthermore, mutations can hitchhike and eventually affect population fitness. We do not attempt to incorporate epistatic effects here but rather investigate the theoretical mechanisms that may allow the persistence of latent genomic fragments in the plasma population despite virus activated from latent cells having a fitness disadvantage. Assuming that latent virus does not have a replicative advantage may lead us to underestimate the survival of latent genomic fragments in the plasma virus population.

Furthermore, if activated virus from latent cells has similar fitness as contemporaneous variants, recombination may not be as important for survival. However, recombination does not merely facilitate survival, but rather allows latent genomic fragments to propagate through the plasma virus population. In the most extreme case where no selection pressure was exerted by the immune response (0 epitopes, S4 Table), approximately 60% of viral sequences contained latent genomic fragments during the last five years of infection in simulations with recombination, while only 6% of sequences were latent in simulations without recombination. While continual recombination with virus circulating in the plasma reduces the size of the genomic fragment inherited from a latent ancestor, even a few latent sites of high replicative fitness may allow the virus to better adapt to different evolutionary pressures. Latency may therefore expand the adaptive potential of HIV-1 besides enabling the virus to hide from immune surveillance and antiretroviral treatment.

Because the patient sequences we chose to study were in envelope, we focused on antibody responses and escape from them. While we did not explicitly account for CTL responses, the epitopes in our model are approximately the same length as CTL epitopes, so our model could be interpreted as one in which CTL responses instead of or in addition to antibodies are providing immune pressure. However, CTL responses exert selection pressure on the whole proteome. Since we only simulated sequence evolution in *env* and ignored the interplay between different genes, our model predictions may not be generalizable to the whole virus. Our future directions include investigating further the balance between replicative fitness and immune escape on the level of the whole virus genome, and adapting our model to simulate HIV-1 evolution and latency under different treatment scenarios.

## Supporting Information

S1 Text The model algorithm. The pseudo-code of our computer simulations, implemented in R.
(DOCX)

S1 Fig The proportion of surviving lineages between samples is similar in phylogenies derived from simulated sequences and from clinical data. (A) Typical phylogeny from our simulations. The proportion of surviving lineages from earlier samples is representative of the tree shape (star- to ladder-like). The legend shows samples through time and the surviving proportion of lineages in parentheses. (B) Green dots represent the proportion of surviving lineages between samples at adjacent time points in clinical data, and black dots represent our simulated data. The stronger green color indicates overlapping data points. The phylogenetic trees were generated from 20 sequences sampled every year per simulation.
(TIF)

S2 Fig Survival of different latent genomic fragments has no effect on diversity for concave fitness maps. We ran 1000 simulations with the concave fitness landscape shown in the insert, and categorized the results based on the number of latent genomic fragments from different origins at 1% or greater frequency at 10 years post-PHI. Increasing the number of latent origins does not increase sequence diversity, which grows linearly regardless of the latent survival.
(TIF)

S3 Fig Number of different latent origins as a function of the proportion of latent lineages in productively infected cells. Envelope indicates the span of 95% of observations, with the mean given by the white line, and the median given by the green line.
(TIF)

S4 Fig The effect of activation rate on the persistence and composition of latent genomic fragments. A) The distribution of the number of different latent origins in productively infected cells as a function of activation rate. B) The distribution of the proportion of virus with latent genomic fragments in productively infected cells as a function of activation rate. 95% envelopes with white lines as the mean and green lines as the median.
(TIF)

S5 Fig The mean sequence divergence and diversity of HIV-1 populations simulated under the alternative model parameters. After approximately 2 years post-PHI, diversity starts to grow linearly in the latent reservoir (blue solid line) while it starts to saturate in plasma (green solid line). Divergence in the latent reservoir (blue dashed line) grows at a slightly slower rate than in plasma (green dashed line).
(TIF)

S6 Fig The effect of recombination on survival of latent lineages under the alternative model parameters. A) Simulations without recombination. B) Simulations with recombination. Grey lines show the proportion of latent lineages in the productively infected cell population of individual simulations, where the bold tan line is the mean proportion of latent lineages and the thin tan lines outline the 95% confidence envelope. Comparing panels A and B clearly shows that recombination facilitates survival of latent forms.
(TIF)

S7 Fig The number of epitopes associated with high fitness cost influences sequence diversity. We compiled the simulation results of varying the number of epitopes between 1 and 15 (S4 Table), and re-categorized them based on the number of epitopes associated with high fitness costs (defined here as fitness costs within 15% of the maximum fitness cost observed in the fitness landscape for a given simulation). Sequence diversity dramatically decreases when the number of epitopes with high fitness costs is either 2 or 3, making escape difficult but not impossible, and is the highest when the number of epitopes is 5 or greater, fully preventing escape from all simultaneous immune responses.
(TIF)

S1 Table Simulation results when the activation rate is varied, with and without recombination. Increasing the activation rate in simulations with recombination increases the proportion of simulations with persistence (≥ 10%) of virus with latent genomic fragments, and the mean proportion of virus sequences with latent genomic fragments. 100 simulations were performed for each activation rate. In simulations without recombination, latent persistence is rare.
(DOC)

S2 Table Simulation results when the recombination rate is varied. Increasing the recombination rate increases the proportion of simulations with persistence (≥ 10%) of virus with latent genomic fragments, and the mean proportion of virus sequences with latent genomic fragments. As the recombination rate increases, the mean number of latent fragments per sequence increases, but the mean size of the fragments decreases. 50 simulations were performed for each recombination rate.
(DOC)

S3 Table Simulation results when the sensitivity of the immune response to new viral antigens in varied, with and without recombination. Increasing the antigen frequency required for eliciting a new immune response in simulations with recombination decreases the survival of virus with latent genomic fragments and sequence diversity while sequence divergence increases. In simulations without recombination, latent persistency is rare. 50 simulations were performed for each value of antigen frequency.
(DOC)

S4 Table Simulation results when the number of epitopes recognized by the immune system is varied, with and without recombination. When the number of epitopes is increased in simulations with recombination, sequence diversity initially decreases, reaching lowest values at 3 and 4 epitopes, and increases thereafter. Sequence divergence follows the reverse trend. Virus with latent genomic fragments rarely survived in simulations without recombination. 50 simulations were performed for each number of epitopes.
(DOC)
