# Supplementary material for: Recombination Enhances HIV-1 Envelope Diversity by Facilitating the Survival of Latent Genomic Fragments in the Plasma Virus Population
Source: PLoS Comput Biol. 2015 Dec 22;11(12):e1004625. doi: 10.1371/journal.pcbi.1004625 (PMC4687844; doi:10.1371/journal.pcbi.1004625)
Supplement: S1 Text — The pseudo-code of our computer simulations, implemented in R. (DOCX) [file pcbi.1004625.s001.docx]

Algorithm

• Initialize cell populations

1. Create founder virus: string of *L* nucleotides (A,U,G,C)

2. Create population of productively infected cells *C* : infect each of *NC* cells with one copy of the founder virus

3. Set tracker for number of generations in latent reservoir to 0 for each of *L*

sites for each of *NC* cells

4. Create latent reservoir: infect each of *NL* resting cells with one copy of the founder virus

5. Set tracker for number of generations in latent reservoir to 0 for each of *NL*

cells

• Initialize selection pressure

1. Set the locations of *NE* non-overlapping, contiguous epitopes of 20 nucleotides in the viral sequence

2. Set the maximum fitness cost associated with recognition at each epitope:

draw from *U[0,0.4]*

3. Choose randomly the locations of the invariant sites in the viral sequence

LOOP: For each generation *t ≤ n.gen*

• Latent reservoir dynamics:

1. For each productively infected cell, draw whether it will move to latent reservoir (probability = *η*)

2. Draw the total number of cells that leave the latent reservoir by activation or death from the binomial distribution: *NLR = binom(1, NL , aL + δ)*

3. Draw from the multinomial distribution how many cells leaving the latent reservoir are activated and how many die:

{*NLA , NLδ } = rmultinom(1, NLR , {aL , δ})*

4. Determine how many cells are proliferated:

*NLP = max{0, N * − (NL − NLR )}*

L

($N_{L}^{*}$ is target size, while *NL* is current size of reservoir)

5. Randomly sample latent reservoir to determine which cells proliferate, activate, or die

6. Remove dead cells from latent reservoir

7. Copy viral sequences of proliferating cells into new cells and add to latent reservoir

8. Remove activated cells and add to population of productively infected cells

9. Update trackers for number of latent generations for latent and productively infected cells

• Productively infected cell dynamics:

1. Mutate cells: Determine the total number of mutations in the cell population, *n.mut = rbinom(1, NC × L, µ).* To efficiently assign mutations, sample *s* = 100, 000 sites in sequence matrix of *NC × L* sites. Distribute *n.mut* mutations across subset s according to nucleotide-specific probabilities of mutation. Draw specific nucleotide substitution at each of *n.mut* sites from nucleotide transition probability matrix (GRT).

2. Produce *P* identical virus from each cell

3. Determine fitness of each virus

a) Determine fitness cost of each antibody *j* at each epitope *i*:

$min(c_{i}^{*},c_{i}^{*}(t-t_{ij}^{0}$)/*d*), where $c_{i}^{*}$ is the maximum fitness cost at epitope *i*, $t_{ij}^{0}$

is the generation at which antibody *j* was introduced, and *d* is the number of

generations required for antibodies to reach full potency

b) For each virus *j*, determine which epitopes are recognized $(\delta_{ij}$) and calculate the maximum fitness cost across all epitopes $c_{j}^{imm}=max\{c_{ij}\delta_{ij}\}$

c) For each virus *j*, determine the number of mutations at invariant sites, $m_{inv}$, and calculate their cost, $c_{j}^{inv}=1-{(1-\psi)}^{m_{inv}}$

d) Calculate the relative cost of each virus, $f_{j}$=(1-$c_{j}^{imm})(1-c_{j}^{inv})$

4. Determine number of dual infections, *ND = rbinom(1, NC , 0.05)*

5. Determine number of dual infections with recombination,

N*R = round (3 × 10−4 × L × ND )*

6. Sample *NC + NR* virus variants based on relative fitness to infect cells

7. Infect *NC* cells with a single virus, add a second virus randomly to *NR* cells

8. Recombine virus in *NR* dually infected cells: In each cell, draw randomly the cross-over position from *U [2, L − 1].* Pick randomly the starting strand. Copy first strand until cross-over position, switch to second strand and copy until the end. Replace the two parent virus strands with the new recombinant.

9. Update the tracker for number of latent generations at each site at each of

*NC* cells

• Immune response: if *t ≥ 30*

1. Draw a random sample *r* = 5000 of productive infected cells

2. At each epitope, determine if there is a new immune response: Calculate the frequency of each unique sequence variant in sample *r*, and determine which epitope sequence variants are at a proportion greater than or equal to *f* . Add epitope sequence variants that are already not in memory to immune response matrix, storing also the current generation.

• Every 30 generations: calculate sequence divergence and diversity of *NC*

productively infected and *NL* latent cells

• Every 300 generations: Randomly store 100 sequences from *NC* productively infected and *NL* latent cells

• *t = t + 1*

END LOOP
