# Supplementary figures and images for: Recombination Enhances HIV-1 Envelope Diversity by Facilitating the Survival of Latent Genomic Fragments in the Plasma Virus Population
Source: PLoS Comput Biol. 2015 Dec 22;11(12):e1004625. doi: 10.1371/journal.pcbi.1004625 (PMC4687844; doi:10.1371/journal.pcbi.1004625)

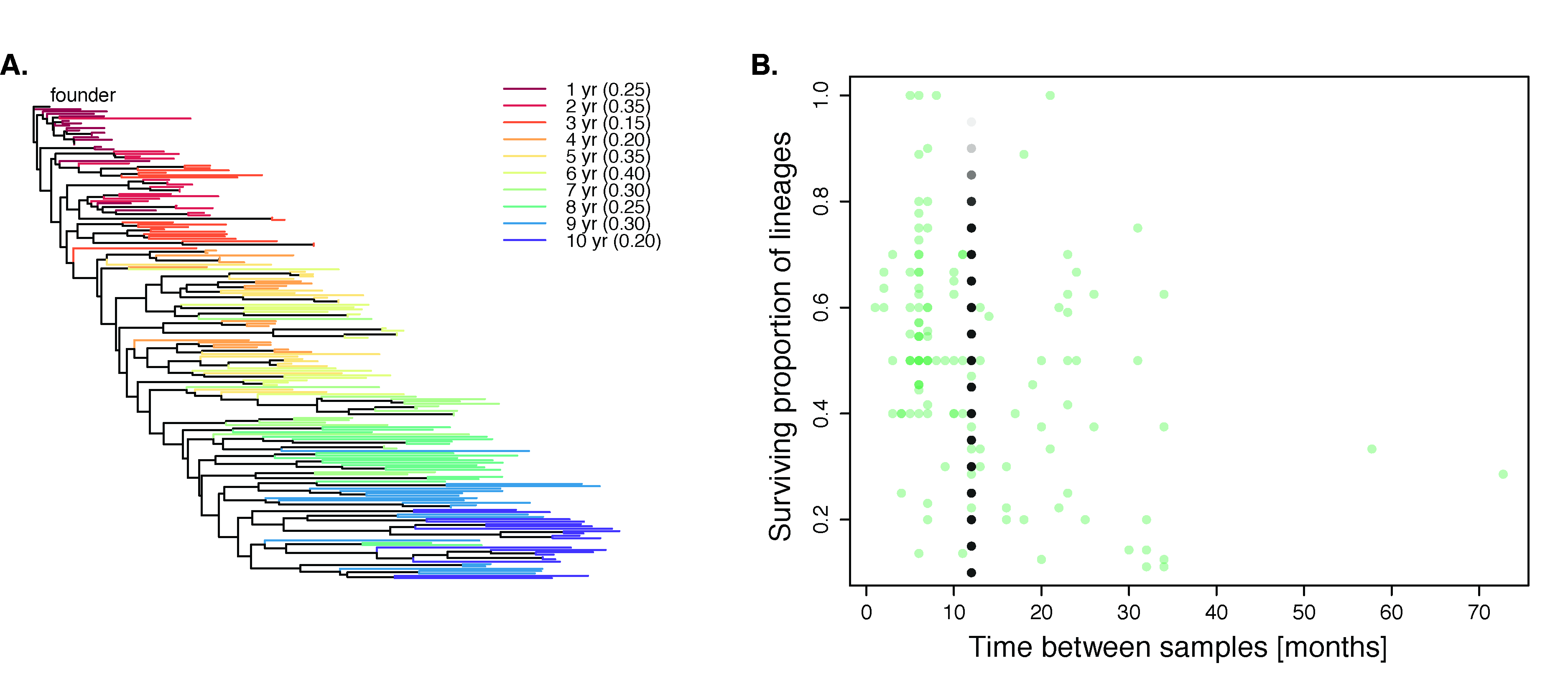

Supplement: S1 Fig — (A) Typical phylogeny from our simulations. The proportion of surviving lineages from earlier samples is representative of the tree shape (star- to ladder-like). The legend shows samples through time and the surviving proportion of lineages in parentheses. (B) Green dots represent the proportion of surviving lineages between samples at adjacent time points in clinical data, and black dots represent our simulated data. The stronger green color indicates overlapping data points. The phylogenetic trees were generated from 20 sequences sampled every year per simulation. (TIF) [file pcbi.1004625.s002.tif]

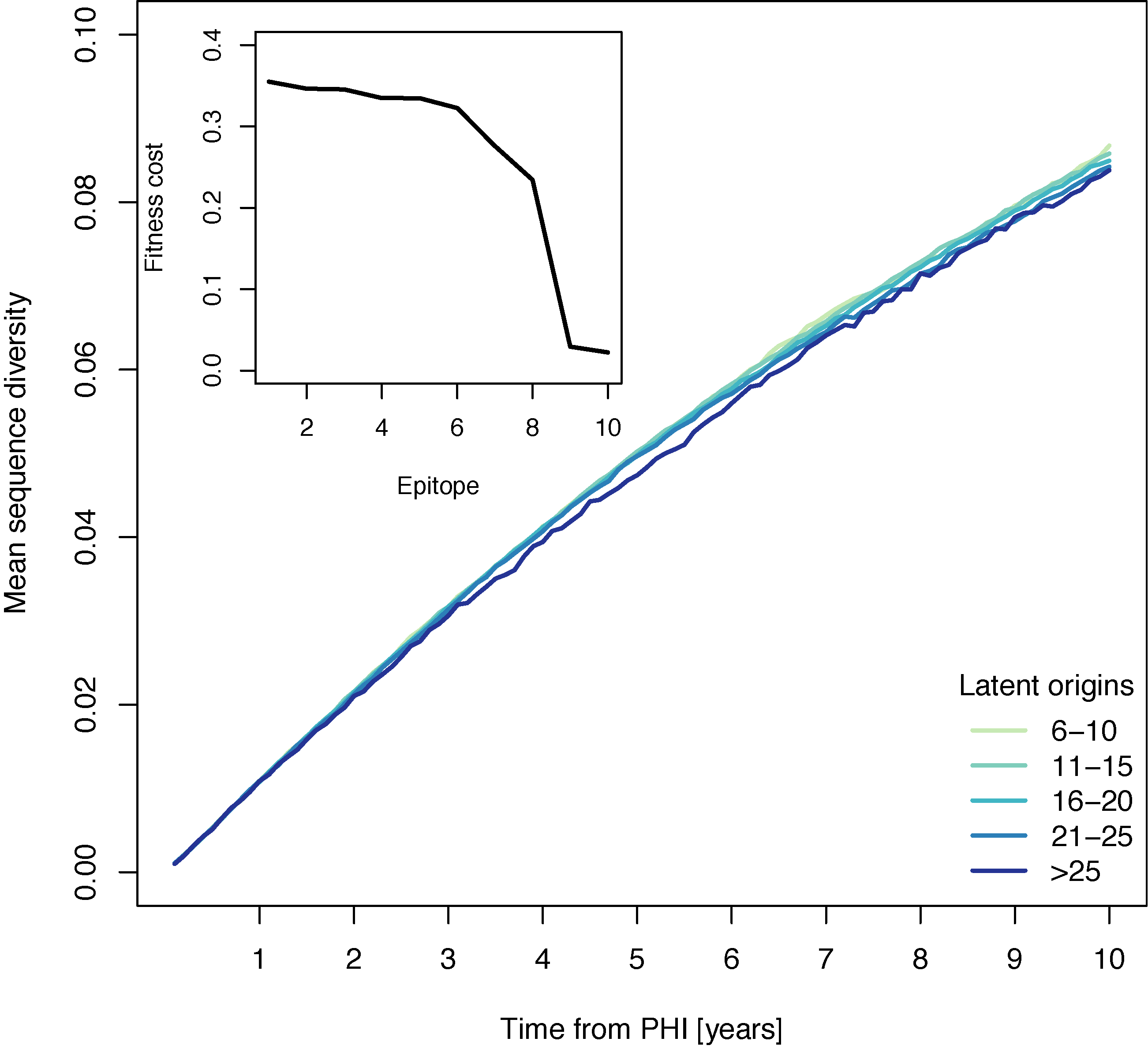

Supplement: S2 Fig — We ran 1000 simulations with the concave fitness landscape shown in the insert, and categorized the results based on the number of latent genomic fragments from different origins at 1% or greater frequency at 10 years post-PHI. Increasing the number of latent origins does not increase sequence diversity, which grows linearly regardless of the latent survival. (TIF) [file pcbi.1004625.s003.tif]

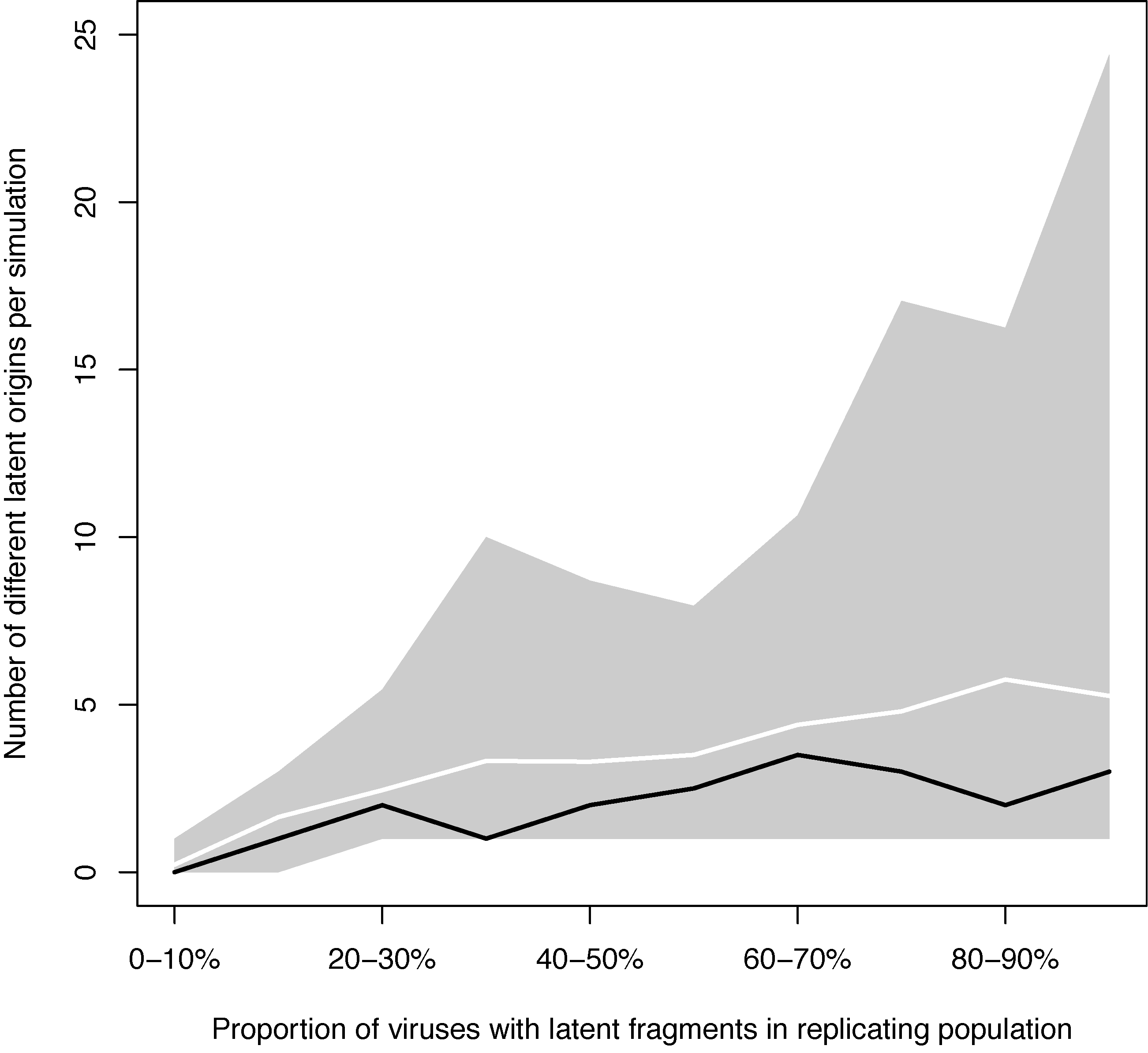

Supplement: S3 Fig — Envelope indicates the span of 95% of observations, with the mean given by the white line, and the median given by the green line. (TIF) [file pcbi.1004625.s004.tif]

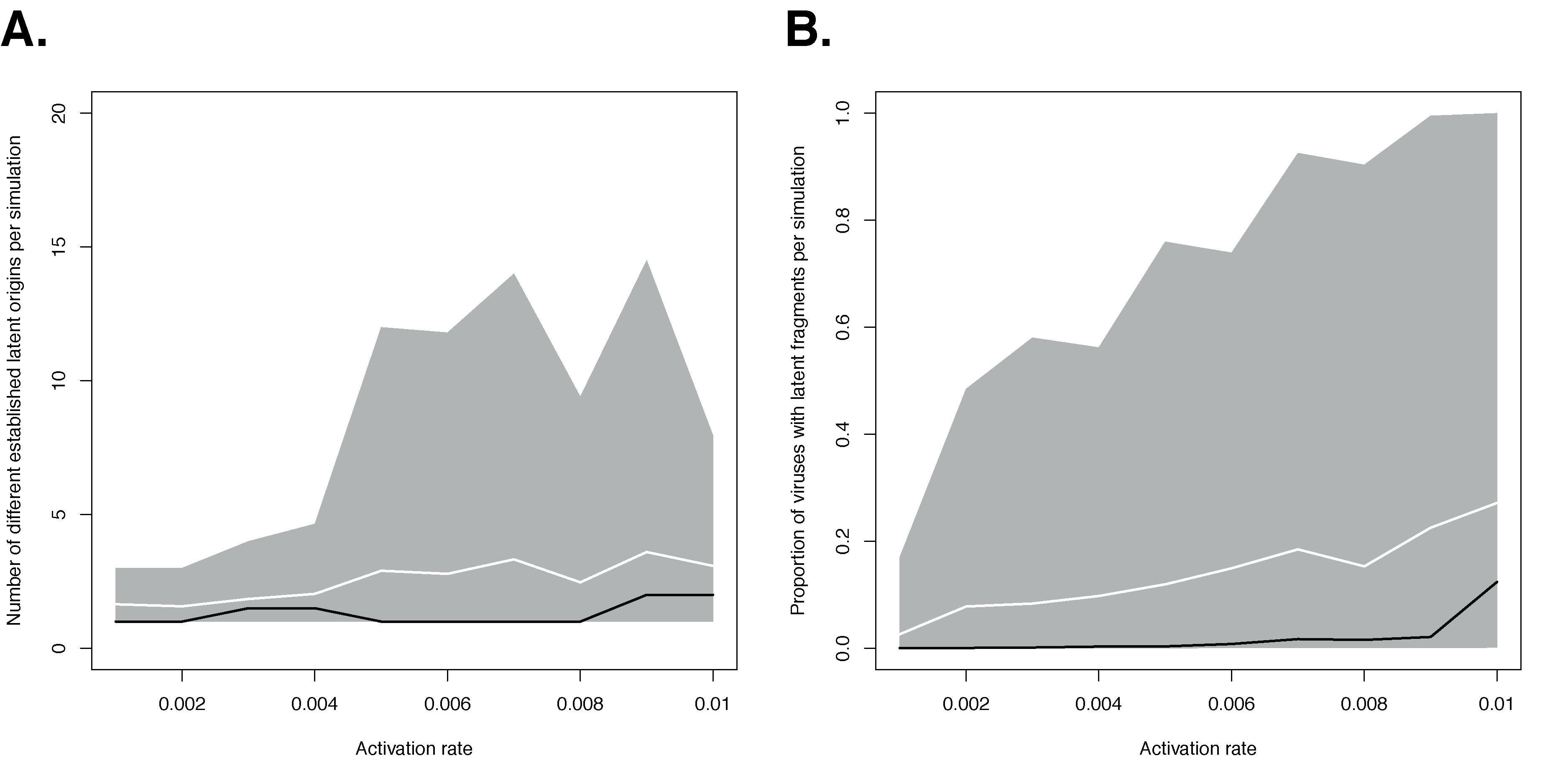

Supplement: S4 Fig — A) The distribution of the number of different latent origins in productively infected cells as a function of activation rate. B) The distribution of the proportion of virus with latent genomic fragments in productively infected cells as a function of activation rate. 95% envelopes with white lines as the mean and green lines as the median. (TIF) [file pcbi.1004625.s005.tif]

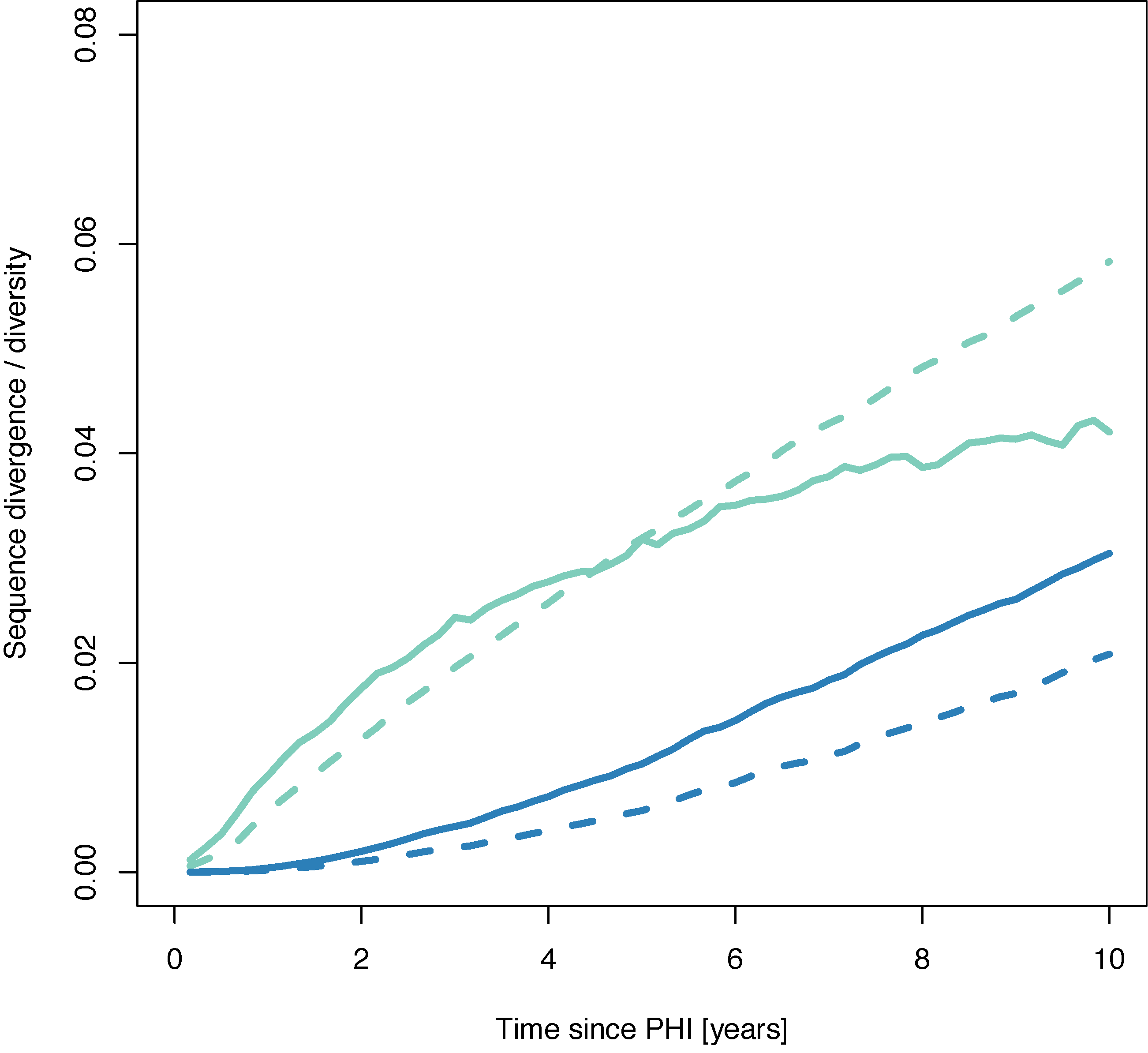

Supplement: S5 Fig — After approximately 2 years post-PHI, diversity starts to grow linearly in the latent reservoir (blue solid line) while it starts to saturate in plasma (green solid line). Divergence in the latent reservoir (blue dashed line) grows at a slightly slower rate than in plasma (green dashed line). (TIF) [file pcbi.1004625.s006.tif]

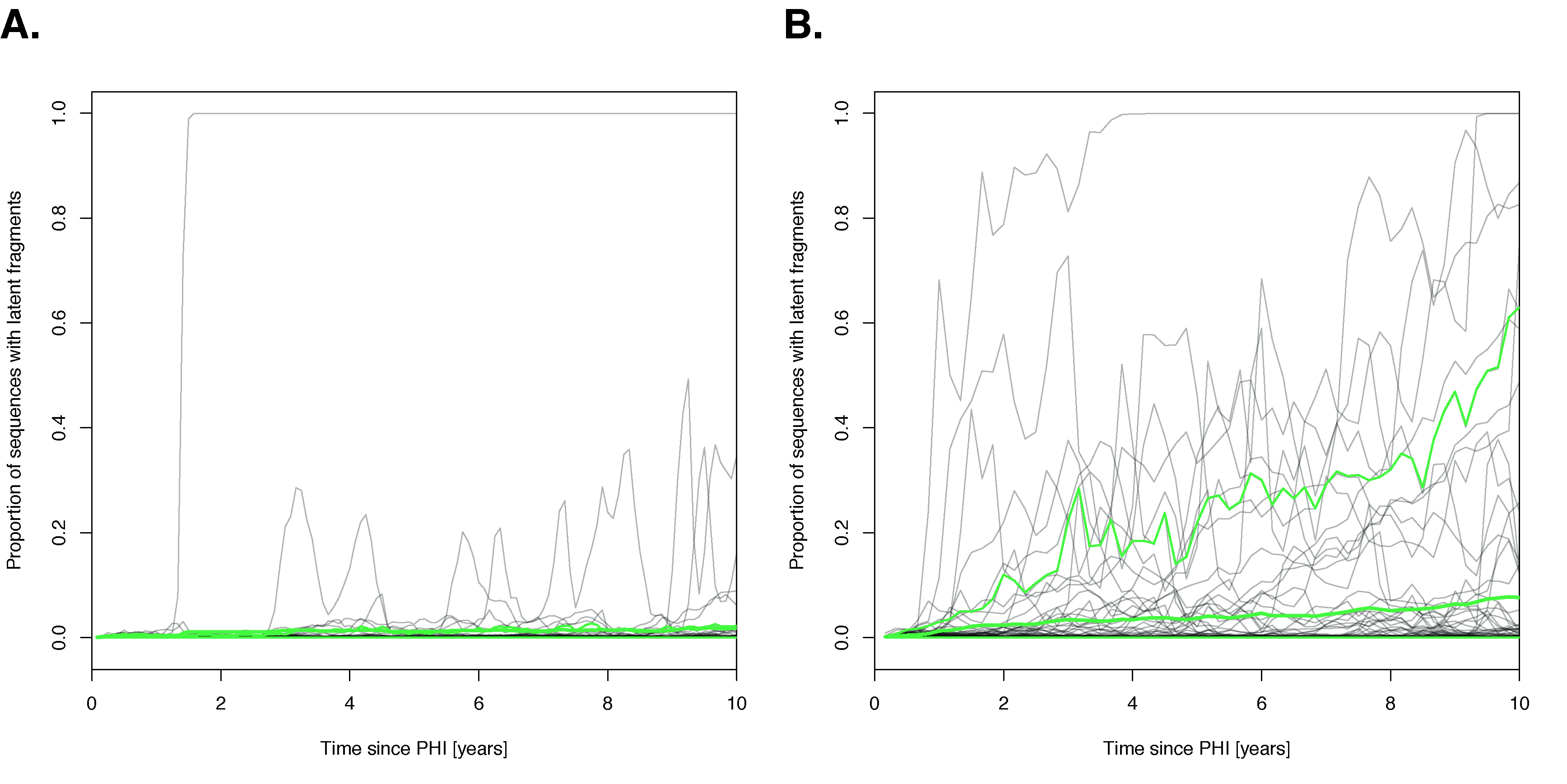

Supplement: S6 Fig — A) Simulations without recombination. B) Simulations with recombination. Grey lines show the proportion of latent lineages in the productively infected cell population of individual simulations, where the bold tan line is the mean proportion of latent lineages and the thin tan lines outline the 95% confidence envelope. Comparing panels A and B clearly shows that recombination facilitates survival of latent forms. (TIF) [file pcbi.1004625.s007.tif]

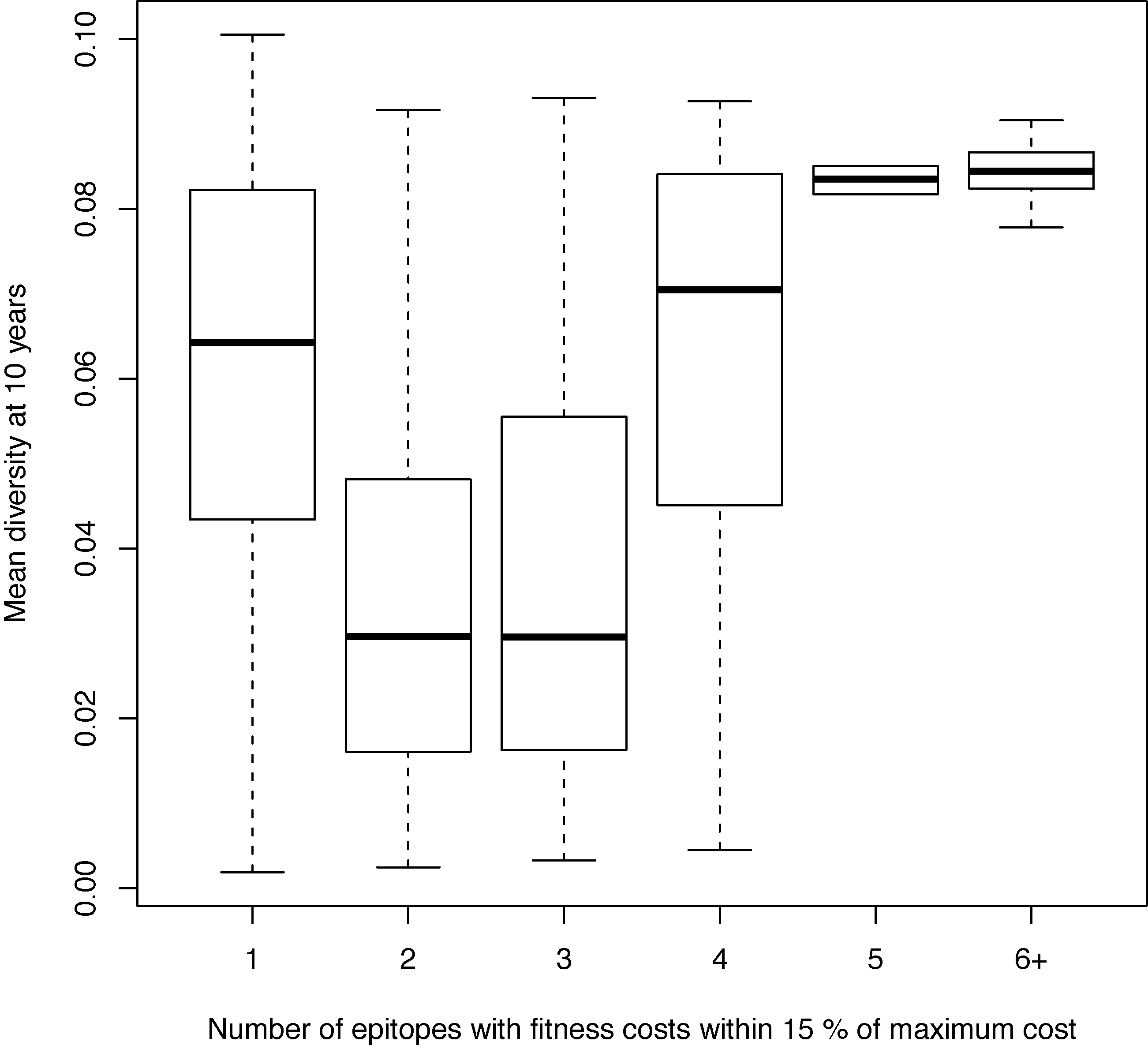

Supplement: S7 Fig — We compiled the simulation results of varying the number of epitopes between 1 and 15 (S4 Table), and re-categorized them based on the number of epitopes associated with high fitness costs (defined here as fitness costs within 15% of the maximum fitness cost observed in the fitness landscape for a given simulation). Sequence diversity dramatically decreases when the number of epitopes with high fitness costs is either 2 or 3, making escape difficult but not impossible, and is the highest when the number of epitopes is 5 or greater, fully preventing escape from all simultaneous immune responses. (TIF) [file pcbi.1004625.s008.tif]
