# Supplementary material for: Recombination Enhances HIV-1 Envelope Diversity by Facilitating the Survival of Latent Genomic Fragments in the Plasma Virus Population
Source: PLoS Comput Biol. 2015 Dec 22;11(12):e1004625. doi: 10.1371/journal.pcbi.1004625 (PMC4687844; doi:10.1371/journal.pcbi.1004625)
Supplement: S1 Table — Increasing the activation rate in simulations with recombination increases the proportion of simulations with persistence (≥ 10%) of virus with latent genomic fragments, and the mean proportion of virus sequences with latent genomic fragments. 100 simulations were performed for each activation rate. In simulations without recombination, latent persistence is rare. (DOC) [file pcbi.1004625.s009.doc]

| Activation rate | Plasma virus with latent genomic fragments | | | Divergence | Diversity |
| --- | --- | --- | --- | --- | --- |
|  | % runs | at 10 y | from 5-10y | at 10 y | at 10 y |
| With recombination | | | | | |
| 0.001 | 9 | 695 (2215) | 395 (1309) | 0.068 (0.011) | 0.049 (0.027) |
| 0.002 | 13 | 1635 (4270) | 1174 (3298) | 0.070 (0.009) | 0.043 (0.026) |
| 0.003 | 17 | 1922 (4076) | 1256 (3007) | 0.069 (0.009) | 0.046 (0.025) |
| 0.004 | 18 | 1988 (4172) | 1468 (3413) | 0.069 (0.010) | 0.041 (0.024) |
| 0.005 | 24 | 2678 (4894) | 1795 (3656) | 0.068 (0.010) | 0.043 (0.024) |
| 0.006 | 25 | 3220 (5245) | 2244 (3963) | 0.069 (0.009) | 0.048 (0.027) |
| 0.007 | 37 | 3811 (5783) | 2775 (4489) | 0.067 (0.010) | 0.047 (0.026) |
| 0.008 | 31 | 3301 (5342) | 2298 (4095) | 0.068 (0.009) | 0.046 (0.025) |
| 0.009 | 40 | 4124 (5951) | 3381 (5026) | 0.069 (0.010) | 0.047 (0.022) |
| 0.01 | 53 | 5273 (6045) | 4077 (5069) | 0.068 (0.008) | 0.051 (0.027) |
| Without recombination | | | | | |
| 0.001 | 0 | 15 (60) | 26 (108) | 0.067 (0.011) | 0.042 (0.029) |
| 0.002 | 1 | 142 (1191) | 125 (1000) | 0.066 (0.009) | 0.040 (0.028) |
| 0.003 | 2 | 362 (1879) | 221 (1507) | 0.067 (0.010) | 0.040 (0.027) |
| 0.004 | 1 | 94 (274) | 89 (253) | 0.067 (0.011) | 0.038 (0.028) |
| 0.005 | 0 | 228 (824) | 110 (264) | 0.066 (0.011) | 0.039 (0.027) |
| 0.006 | 4 | 431 (2063) | 296 (984) | 0.066 (0.011) | 0.041 (0.027) |
| 0.007 | 2 | 424 (2118) | 398 (2104) | 0.068 (0.009) | 0.042 (0.027) |
| 0.008 | 3 | 571 (2053) | 286 (888) | 0.066 (0.009) | 0.043 (0.027) |
| 0.009 | 4 | 615 (2206) | 428 (1539) | 0.067 (0.010) | 0.042 (0.028) |
| 0.01 | 3 | 435 (2077) | 270 (1216) | 0.067 (0.009) | 0.039 (0.026) |
