# Supplementary material for: Recombination Enhances HIV-1 Envelope Diversity by Facilitating the Survival of Latent Genomic Fragments in the Plasma Virus Population
Source: PLoS Comput Biol. 2015 Dec 22;11(12):e1004625. doi: 10.1371/journal.pcbi.1004625 (PMC4687844; doi:10.1371/journal.pcbi.1004625)
Supplement: S2 Table — Increasing the recombination rate increases the proportion of simulations with persistence (≥ 10%) of virus with latent genomic fragments, and the mean proportion of virus sequences with latent genomic fragments. As the recombination rate increases, the mean number of latent fragments per sequence increases, but the mean size of the fragments decreases. 50 simulations were performed for each recombination rate. (DOC) [file pcbi.1004625.s010.doc]

| Recombination | Plasma virus with latent genomic fragments | | | Divergence | Diversity | No. (size) |
| --- | --- | --- | --- | --- | --- | --- |
|  | % runs | at 10 y | from 5-10y | at 10 y | at 10 y | fragments at 10 y |
| 0.1 | 6 | 559 (2047) | 295 (840) | 0.067 (0.009) | 0.043 (0.026) | 1.1 (346) |
| 0.16 | 4 | 421 (1537) | 286 (959) | 0.067 (0.009) | 0.046 (0.026) | 1.1 (325) |
| 0.25 | 16 | 1984 (4712) | 1511 (3771) | 0.066 (0.009) | 0.044 (0.028) | 1.2 (215) |
| 0.40 | 14 | 1602 (3471) | 1016 (2718) | 0.068 (0.009) | 0.049 (0.027) | 1.3 (100) |
| 0.63 | 16 | 1847 (4047) | 1152 (2563) | 0.071 90.011) | 0.037 (0.023) | 1.6 (64) |
| 1.0 | 28 | 2837 (5016) | 1948 (3652) | 0.068 (0.010) | 0.044 (0.025) | 1.6 (76) |
| 1.6 | 28 | 2599 (4752) | 2162 (4172) | 0.069 (0.010) | 0.045 (0.023) | 1.5 (35) |
| 2.5 | 44 | 5031 (5876) | 3380 (4529) | 0.069 (0.009) | 0.060 (0.024) | 1.8 (27) |
| 4.0 | 54 | 6765 (6089) | 4700 (5056) | 0.073 (0.007) | 0.063 (0.025) | 1.9 (14) |
| 6.3 | 74 | 9365 (6450) | 8605 (6217) | 0.074 (0.010) | 0.075 (0.020) | 3.9 (9) |
| 10 | 94 | 12045 (4106) | 10425 (4460) | 0.079 (0.009) | 0.091 (0.023) | 3.6 (6) |
