# Supplementary material for: Recombination Enhances HIV-1 Envelope Diversity by Facilitating the Survival of Latent Genomic Fragments in the Plasma Virus Population
Source: PLoS Comput Biol. 2015 Dec 22;11(12):e1004625. doi: 10.1371/journal.pcbi.1004625 (PMC4687844; doi:10.1371/journal.pcbi.1004625)
Supplement: S3 Table — Increasing the antigen frequency required for eliciting a new immune response in simulations with recombination decreases the survival of virus with latent genomic fragments and sequence diversity while sequence divergence increases. In simulations without recombination, latent persistency is rare. 50 simulations were performed for each value of antigen frequency. (DOC) [file pcbi.1004625.s011.doc]

| Antigen frequency | Plasma virus with latent genomic fragments | | | Divergence | Diversity |
| --- | --- | --- | --- | --- | --- |
| (%) | % runs | at 10 y | from 5-10y | at 10 y | at 10 y |
| With recombination | | | | | |
| -3.0 | 68 | 6172 (5273) | 4490 (3830) | 0.060 (0.009) | 0.067 (0.020) |
| -2.8 | 50 | 5191 (5755) | 3699 (4450) | 0.061 (0.010) | 0.064 (0.022) |
| -2.6 | 50 | 4437 (5230) | 3380 (4174) | 0.062 (0.010) | 0.057 (0.026) |
| -2.4 | 36 | 3571 (5146) | 2209 (3278) | 0.064 (0.011) | 0.056 (0.025) |
| -2.2 | 35 | 3334 (5166) | 2527 (4059) | 0.066 (0.009) | 0.049 (0.028) |
| -2.0 | 28 | 2837 (5016) | 1948 (3652) | 0.068 (0.010) | 0.044 (0.025) |
| -1.8 | 24 | 2747 (5357) | 2107 (4203) | 0.074 (0.011) | 0.034 (0.023) |
| -1.6 | 20 | 2641 (5453) | 2161 (4601) | 0.074 (0.006) | 0.031 (0.022) |
| -1.4 | 10 | 1785 (4285) | 982 (2781) | 0.077 (0.008) | 0.031 (0.021) |
| -1.2 | 16 | 2459 (5519) | 1963 (4592) | 0.077 (0.009) | 0.026 (0.017) |
| -1.0 | 10 | 1921 (4855) | 1256 (3702) | 0.077 (0.009) | 0.032 (0.021) |
| Without recombination | | | | | |
| -3.0 | 6 | 513 (1377) | 340 (446) | 0.058 (0.008) | 0.061 (0.026) |
| -2.8 | 6 | 875 (2144) | 593 (1354) | 0.059 (0.010) | 0.059 (0.028) |
| -2.6 | 4 | 167 (554) | 160 (504) | 0.061 (0.008) | 0.054 (0.027) |
| -2.4 | 6 | 889 (3117) | 730 (2924) | 0.063 (0.011) | 0.049 (0.027) |
| -2.2 | 2 | 184 (441) | 162 (408) | 0.064 (0.009) | 0.044 (0.027) |
| -2.0 | 0 | 338 (1101) | 166 (352) | 0.0676(0.012) | 0.045 (0.028) |
| -1.8 | 4 | 293 (1058) | 155 (545) | 0.069 (0.010) | 0.036 (0.026) |
| -1.6 | 6 | 972 (3588) | 478 (1700) | 0.072 (0.011) | 0.032 (0.025) |
| -1.4 | 2 | 322 (2113) | 106 (434) | 0.071 (0.010) | 0.027 (0.022) |
| -1.2 | 0 | 22 (62) | 20 (44) | 0.075 (0.009) | 0.024 (0.020) |
| -1.0 | 2 | 413 (2206) | 284 (1613) | 0.075 (0.010) | 0.020 (0.015) |
