# Supplementary material for: Recombination Enhances HIV-1 Envelope Diversity by Facilitating the Survival of Latent Genomic Fragments in the Plasma Virus Population
Source: PLoS Comput Biol. 2015 Dec 22;11(12):e1004625. doi: 10.1371/journal.pcbi.1004625 (PMC4687844; doi:10.1371/journal.pcbi.1004625)
Supplement: S4 Table — When the number of epitopes is increased in simulations with recombination, sequence diversity initially decreases, reaching lowest values at 3 and 4 epitopes, and increases thereafter. Sequence divergence follows the reverse trend. Virus with latent genomic fragments rarely survived in simulations without recombination. 50 simulations were performed for each number of epitopes. (DOC) [file pcbi.1004625.s012.doc]

| No. epitopes | Plasma virus with latent genomic fragments | | | Divergence | Diversity |
| --- | --- | --- | --- | --- | --- |
|  | % runs | at 10 y | from 5-10y | at 10 y | at 10 y |
| With recombination | | | | | |
| 0 | 100 | 11446 (2299) | 8685 (2316) | 0.047 (0.002) | 0.081 (0.003) |
| 1 | 28 | 3121 (4603) | 2158 (3709) | 0.057 (0.005) | 0.081 (0.012) |
| 2 | 26 | 2148 (3926) | 1519 (2706) | 0.062 (0.007) | 0.064 (0.021) |
| 3 | 18 | 1638 (4191) | 1431 (3875) | 0.071 (0.008) | 0.045 (0.026) |
| 4 | 14 | 1669 (4304) | 1300 (3782) | 0.071 (0.009) | 0.033 (0.025) |
| 5 | 12 | 1515 (4145) | 1364 (3892) | 0.072 (0.008) | 0.035 (0.025) |
| 6 | 6 | 726 (2209) | 466 (1466) | 0.070 (0.009) | 0.041 (0.024) |
| 7 | 34 | 3278 (5111) | 2506 (4050) | 0.070 (0.009) | 0.041 (0.027) |
| 8 | 8 | 781 (2235) | 566 (1831) | 0.071 (0.008) | 0.040 (0.036) |
| 9 | 32 | 3925 (5942) | 2438 (4936) | 0.069 (0.009) | 0.043 (0.025) |
| 10 | 28 | 2837 (5016) | 1948 (3652) | 0.068 (0.010) | 0.044 (0.025) |
| 11 | 20 | 2437 (4583) | 1449 (2841) | 0.068 (0.008) | 0.049 (0.026) |
| 12 | 22 | 2184 (4170) | 1479 (3186) | 0.069 (0.009) | 0.050 (0.024) |
| 13 | 34 | 3976 (5985) | 2697 (4074) | 0.068 (0.009) | 0.053 (0.025) |
| 14 | 46 | 4075 (5220) | 3163 (3847) | 0.064 (0.008) | 0.058 (0.023) |
| 15 | 34 | 3064 (4441) | 2015 (3265) | 0.067 (0.010) | 0.056 (0.029) |
| Without recombination | | | | | |
| 0 | 24 | 1250 (971) | 948 (726) | 0.047 (0.003) | 0.080 (.004) |
| 1 | 6 | 417 (815) | 330 (611) | 0.055 (0.0050 | 0.078 (0.012) |
| 2 | 0 | 135 (439) | 80 (162) | 0.064 (0.007) | 0.053 (0.025) |
| 3 | 0 | 34 (108) | 35 (89) | 0.068 (0.009) | 0.039 (0038) |
| 4 | 0 | 8 (17) | 32 (102) | 0.079 (0.01) | 0.025 (0.023) |
| 5 | 4 | 489 (2367) | 191 (826) | 0.069 (0.008) | 0.030 (0.023) |
| 6 | 2 | 722 (2974) | 666 (2954) | 0.068 (0.008) | 0.032 (0.023) |
| 7 | 0 | 43 (129) | 26 (48) | 0.070 (0.009) | 0.034 (0.028) |
| 8 | 0 | 36 (69) | 43 (81) | 0.069 (0.011) | 0.033 (0.027) |
| 9 | 2 | 148 (890) | 123 (542) | 0.068 (0.010) | 0.029 (0.025) |
| 10 | 0 | 338 (1101) | 166 (352) | 0.0676(0.012) | 0.045 (0.028) |
| 11 | 2 | 186 (401) | 178 (428) | 0.065 (0.008) | 0.052 (0.030) |
| 12 | 4 | 383 (2120) | 356 (1866) | 0.066 (0.009) | 0.042 (0.027) |
| 13 | 6 | 418 (2134) | 448 (2058) | 0.066 (0.008) | 0.048 (0.025) |
| 14 | 4 | 195 (879) | 168 (677) | 0.065 (0.008) | 0.043 (0.027) |
| 15 | 4 | 591 (2307) | 306 (1090) | 0.064 (0.010) | 0.056 (0.029) |
